# Commercial washing and storage over shelf life impact bacterial communities more than the fungal communities on baby spinach

**DOI:** 10.1128/spectrum.04007-25

**Published:** 2026-06-15

**Authors:** Tamara Walsky, Sriya Sunil, Samantha Bolten, Renata Ivanek, Martin Wiedmann

**Affiliations:** 1 Department of Food Science, Cornell University 5922, Ithaca, New York, USA; 2 Department of Population Medicine and Diagnostic Sciences, Cornell University, Ithaca, New York, USA; The Pennsylvania State University, University Park, Pennsylvania, USA

**Keywords:** spoilage, produce, 16S rRNA and ITS amplicon sequencing, baby spinach

## Abstract

Many types of minimally processed fresh produce, including baby spinach, are sensitive to microbial spoilage and other defects that influence quality and consumer acceptance. However, there is limited understanding of how various growing, processing, and storage practices can influence the composition of bacterial and fungal communities on leafy greens, including the proliferation of spoilage microorganisms. Baby spinach samples obtained from a single supply chain at various stages of the produce supply chain (i.e., after harvest and after commercial washing, packaging, and storage) over 1 year were analyzed using 16S ribosomal RNA and internal transcribed spacer amplicon sequencing to assess changes in the bacterial and fungal microbiota. Our data showed that there were significant differences in the bacterial and fungal community composition of Harvest samples associated with (i) the geographic area where the spinach was grown and (ii) seasonality (i.e., time since the start of the growing season). After commercial washing and during storage, *Pseudomonas* spp. dominated the bacterial community (>60% average relative abundance [RA], compared to 8.62% average RA on Harvest samples). Analysis at the amplicon sequence variant (ASV) level revealed that the RA of 39 out of 868 *Pseudomonas* ASVs significantly increased over shelf life, indicating that only select taxa from a particular genus might drive spoilage. Four of the top five fungal genera maintained a high RA across harvest, washing, and postharvest storage, except for one genus (*Vishniacozyma*) that was only represented among the top five in samples after washing. Our data could be used to inform more targeted quality management practices.

**IMPORTANCE:**

While spoilage of leafy greens can be influenced by a variety of factors, the limited understanding of how microbial communities change throughout the various stages of the produce supply chain remains a barrier to identifying factors that drive baby spinach spoilage. Additionally, many studies focus solely on the bacterial community, leaving gaps in understanding how the fungal community is impacted by processing and storage conditions. The findings of this study deepen our understanding of how the bacterial and fungal communities on baby spinach are influenced by both (i) preharvest factors (i.e., growing region and seasonality) and (ii) postharvest factors (i.e., commercial washing/packaging and storage over shelf life). This research deepens our understanding of the variation of the microbial composition of leafy greens and specifically suggests the potential value of spoilage mitigation efforts that target specific *Pseudomonas* taxa.

## INTRODUCTION

The fresh produce industry is motivated to reduce quality defects and premature spoilage. Spoilage of leafy greens, characterized by changes in consistency, texture, and color (e.g., discoloration, sliming, and wilting), can be influenced by several factors, including physical damage from commercial processing and shipping (1), and distribution and storage conditions (2), which could potentially facilitate the proliferation of microorganisms (3, 4). Predicting spoilage of leafy greens is difficult, with a need for improved data on the key microbial taxa that are involved in the process, which could facilitate future work on mechanisms contributing to spoilage. Baby spinach is the second most produced leafy green in the United States, behind lettuce (5), and was selected here as a model to study microbial spoilage of fresh produce. Production of baby spinach occurs year-round in the United States, but in different areas (primarily either in the Salinas, CA and Yuma, AZ areas) (6–8), which could impact the baby spinach microbiome. After harvest, baby spinach is typically subjected to commercial washing (e.g., in chlorinated water), packaging, and postharvest refrigerated storage. Exploring how shifts in baby spinach microbiota are impacted by commercial growing, harvesting, processing/packaging, and storage practices may identify opportunities to address spoilage and more accurately predict shelf life.

While many studies have used culture-dependent methods to quantify microbial populations on leafy greens, culture-independent methods, such as 16S ribosomal RNA (rRNA) gene amplicon sequencing, can provide for more comprehensive insight into the composition of microbial populations. While a number of studies have assessed bacterial communities on leafy greens (1, 9–12), few have examined fungal communities (13, 14). In addition, few studies have comprehensively evaluated microbial population dynamics in produce using a high-resolution longitudinal study design from primary production to the end of shelf life; previous studies often have only collected a few samples throughout a calendar year or season (9–11, 15). This leaves gaps in understanding the changes in fungal and bacterial communities and their impact on spoilage and quality. In addition, previous studies characterizing leafy green microbial communities often reported results in higher taxonomic levels, such as family or order (1, 16), which is unlikely to fully capture community dynamics. Identifying the dynamics of taxa (i.e., amplicon sequencing variant [ASV] or operational taxonomic unit [OTU]) can offer higher resolution insights on community dynamics (e.g., identification of specific taxa that drive spoilage) and may reveal opportunities for targeted management practices to mitigate spoilage and improve the quality of baby spinach and other leafy greens.

## RESULTS

### Mock sample results confirm that the methods used can reliably recover bacterial diversity

Three out of eight genera included in the 16S rRNA gene amplicon sequencing positive control (i.e., *Listeria, Pseudomonas,* and *Bacillus*) were detected in each of the seven sequencing runs at relative abundances consistent with the expected relative abundance for these genera in the mock community (17) (see Table S1 for details). *Salmonella* and *Escherichia* (present in the mock community at approximately 0.1%) were detected in 6/7 sequencing runs, while *Lactobacillus* (present in the mock community at approximately 0.01%) was detected in 0/7 sequencing runs. These data suggest a detection limit of 0.1%, consistent with the detection limit reported by the manufacturer of the mock community (17).

### Both harvest area and time from the beginning of the growing season impact bacterial diversity in Harvest samples

**Fig 1:**
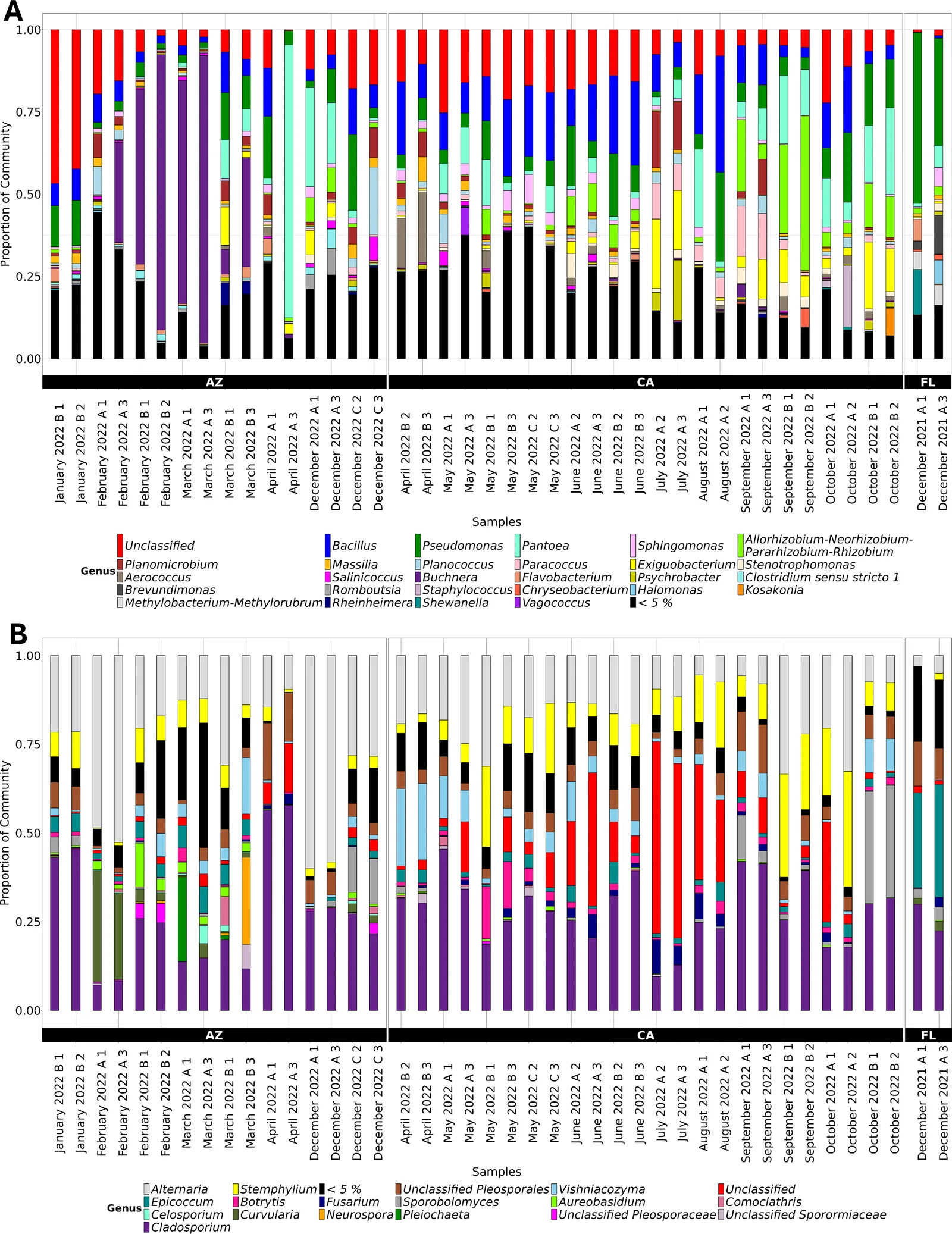
The relative abundance of bacterial (**A**) and fungal (**B**) groups in 21 Harvest samples as determined by 16S rRNA and internal transcribed spacer amplicon sequencing. Data for duplicates tested for each sample are shown. Sample name is coded as follows: “January 2022” = Month and year; “B” = Lot B; and “1” = replicate (replicate 1). Samples are grouped by area, as indicated by the facet labels AZ (Arizona), CA (California), and FL (Florida). All genera with an average relative abundance of less than 5% are grouped into their own category. Further information on the taxa listed can be obtained from the NCBI taxonomy browser at https://www.ncbi.nlm.nih.gov/datasets/taxonomy/tree/.

Across all 21 Harvest samples tested, 676 bacterial groups were identified, representing 675 taxa (from the “genus” field in the taxonomic table produced by SILVA v.138.1; see Table S2 for a full list) as well as a single group that included all Unclassified ASVs; the term “bacterial groups” is used to refer to both the 675 taxa and the category “Unclassified ASVs” as some of these taxa do not represent recognized genera (see Table S2 for details). A total of 28 bacterial groups showed a relative abundance of >5% in at least one of the two replicates for at least one of the 21 samples (Fig. 1A). The bacterial groups with the highest average relative abundance in Harvest samples from the Salinas, CA, and Yuma, AZ areas included *Bacillus*, *Buchnera*, Unclassified ASVs, *Pantoea*, and *Pseudomonas* (see Table 1 and Table S3 for details). Relative abundances ranged widely between samples (Fig. 1A); for example, the relative abundance of unclassified bacterial ASVs ranged from 0.51% to 45.5%.

**TABLE 1:** The top five bacterial and fungal groups in terms of average relative abundance of all Salinas, CA, and Yuma, AZ, area samples at Harvest, Day Initial, and two key days of shelf life

Rank	Harvest (Yuma, AZ/Salinas, CA areas)^*a*^^,*b*^	Day Initial^*a*^	Day 7^*a*^	Day 21/22^*a*^
Bacterial groups				
1	*Buchnera/Bacillus* (22.8%/14.8%)	*Pseudomonas* (61.4%)	*Pseudomonas* (74.6%)	*Pseudomonas* (65.8%)
2	Unclassified ASVs (13.8%/12.5%)	*Pantoea* (13.7%)	*Pantoea* (7.0%)	*Flavobacterium* (11.1%)
3	*Pantoea/Pseudomonas* (9.9%/9.1%)	*Psychrobacter* (4.2%)	*Shewanella* (3.6%)	*Shewanella* (3.9%)
4	*Pseudomonas/Pantoea* (7.9%/8.4%)	*Exiguobacterium* (3.5%)	*Psychrobacter* (2.8%)	*Pantoea* (3.6%)
5	*Bacillus/Allorhizobium-Neorhizobium-Pararhizobium-Rhizobium* (6.4%/7.4%)	*Bacillus* (2.7%)	*Erwinia* (2.5%)	*Sphingobacterium* (2.3%)
Sum of top five groups	60.8%/52.2%	85.5%	90.5%	86.7%
Fungal groups				
1	*Alternaria/Cladosporium* (28.1%/28.3%)	*Cladosporium* (36.0%)	*Cladosporium* (31.2%)	*Cladosporium* (33.6%)
2	*Cladosporium/Alternaria* (27.2%/16.2%)	*Alternaria* (20.9%)	*Vishniacozyma* (23.2%)	*Vishniacozyma* (23.7%)
3	Unclassified *Pleosporales/*Unclassified OTUs (5.4%/13.7%)	*Vishniacozyma* (13.5%)	*Alternaria* (18.5%)	*Alternaria* (16.8%)
4	*Stemphylium* (4.7%/12.0%)	Unclassified *Pleosporales* (7.1%)	Unclassified *Pleosporales* (6.4%)	Unclassified *Pleosporales* (9.8%)
5	*Curvularia/Vishniacozyma* (4.6%/6.0%)	*Stemphylium* (7.1%)	*Stemphylium* (5.3%)	*Stemphylium* (6.7%)
Sum of top five groups	70.0%/76.2%	84.6%	84.6%	90.6%

^*a*^ These percentages were calculated using all samples from the Yuma, AZ and Salinas, CA areas; Southeast USA samples (Florida [FL] and Georgia [GA]) are not included in the data shown here.

^*b*^ As the bacterial and fungal community compositions of Harvest samples are significantly different by area via PERMANOVA (*P*< 0.05), the top five groups for each area and the corresponding relative abundance are listed separately.

For Harvest samples, use of a mixed effects model (as implemented in MaAsLin2 [18]) that accounts for the impact of area and the days from the start of the growing season on the differential abundance of bacterial groups identified (i) 33 groups differentially abundant by area and (ii) 30 groups differentially abundant by time from the start of the growing season (Tables S4 and S5). Among the groups differentially abundant by area, 13 showed higher relative abundance in the Salinas, CA area, including 5 groups that had an average relative abundance >1% (e.g., *Bacillus*; see Table S4). The 20 groups with a significantly higher relative abundance in the Yuma, AZ area samples include 5 with an average relative abundance >1% (e.g., *Buchnera*; see Table S4). All 30 groups that showed differential abundance by time from the start of the growing season showed a decrease in relative abundance over the course of the season (Table S5); the largest decrease was found for *Ralstonia* (log_2_ fold change of −11.3 over the season). The majority of the groups with differential abundance over the course of the season (25/30) showed <1% average relative abundance in both the first and second half of the growing season (Table S5); five groups (e.g., *Sphingomonas*) showed relative abundance >1% in the first half of the season.

**Fig 2:**
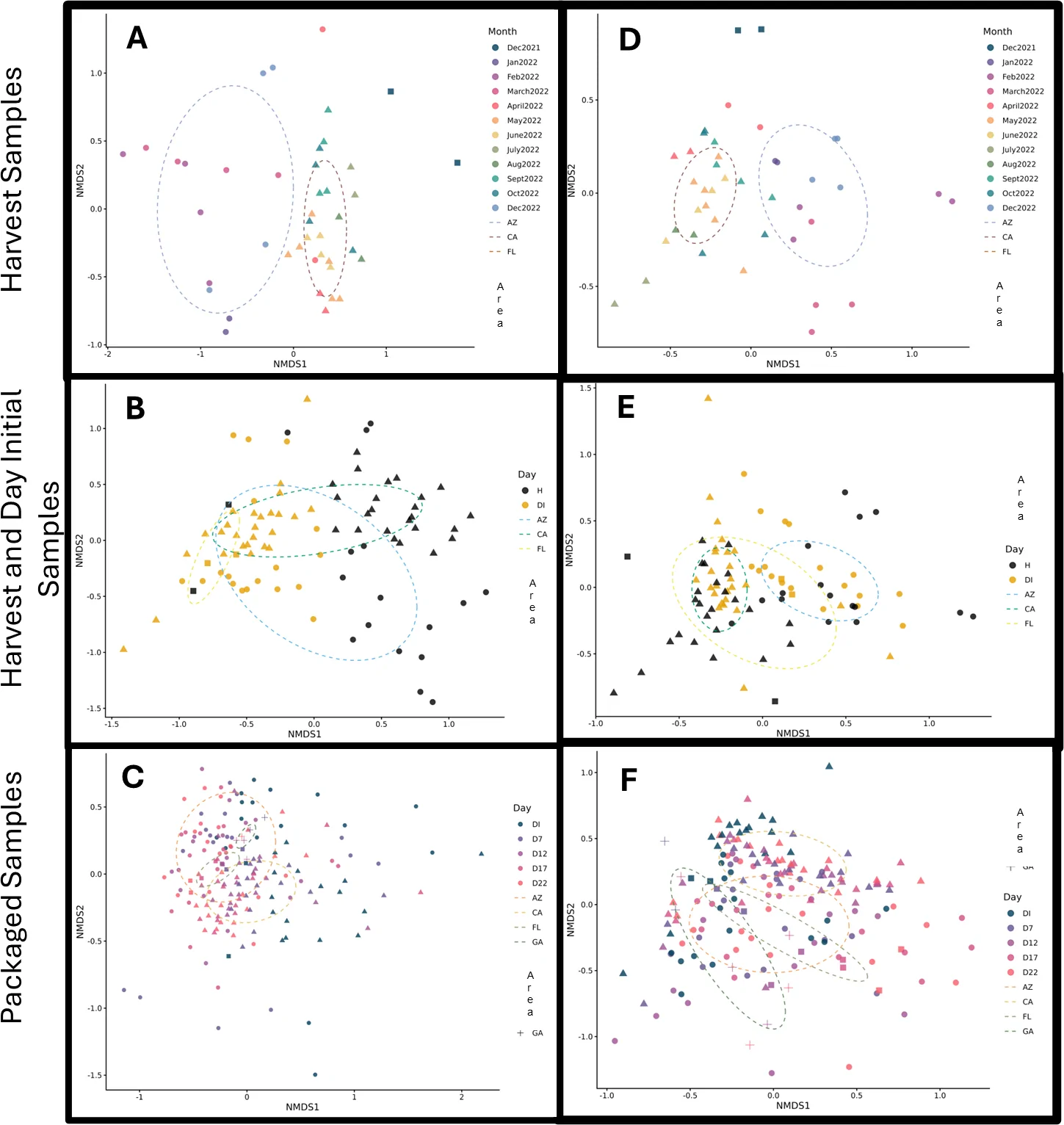
Non-metric multidimensional scaling (NMDS) plots showing compositional dissimilarities of bacterial communities (**A–C**) and fungal communities (**D–F**) on baby spinach at different supply chain stages. These NMDS plots utilize Bray-Curtis values from the 16S rRNA ASV and internal transcribed spacer amplicon OTU sequencing count data. (**A and D**) Harvest samples across different growing areas; different colors denote months that the samples were grown (e.g., January and February). (**B and E**) Harvest and Day Initial samples across different growing areas; different colors denote H (Harvest) and DI (Day Initial). Samples designated as DI represent the leafy greens collected after washing in the processing facility and short post-processing storage while being shipped to Ithaca, NY. (**C and F**) Packaged samples (5-day interval samples only) across different growing areas; different colors denote day of shelf life (e.g., Day Initial). Across all panels, different shapes denote the area where spinach samples were grown (AZ, Yuma, AZ area; CA, Salinas, CA area; FL, Florida; and GA, Georgia), and ellipses denote 95% confidence levels for a multivariate *t*-distribution around the ordinated data points.

Nonmetric multidimensional scaling (NMDS) assembled Salinas, CA area samples separately and more tightly than Yuma, AZ area samples (Fig. 2A), supported by a significant PERMDISP (*P* = 0.01; see Table S6). A PERMANOVA supported that ASVs were significantly different between harvest area (*P* = 0.001, *R*^2^ = 0.2; see Table S6). We acknowledge that the significant PERMDISP results confirm that the smaller group (i.e., Harvest samples from the Yuma, AZ area) has a larger dispersion, which could result in a liberal PERMANOVA result (19), suggesting that the low *P*-value should be interpreted with caution. We still elected to use a PERMANOVA over ANOSIM and Mantel tests, as it has been shown to be relatively less liberal (19).

Among the three bacterial diversity metrics calculated for Harvest samples, Pielou’s Evenness differed significantly (*P* < 0.05; *t*-test) between samples from the Salinas, CA area and Yuma, AZ area (*J* values of 0.77 and 0.65, respectively, see Table S7). The other two bacterial diversity metrics (Richness and Shannon indices) did not differ significantly between these areas. A Spearman rank correlation test also indicated negative correlations (*ρ* = −0.74 and −0.65, respectively, *P* < 0.05) between the number of days after the start of the growing season and two of the three diversity metrics evaluated (i.e., Richness and Shannon indices), suggesting a significant decline in bacterial diversity toward the end of the growing season.

### Compared to Harvest samples, Day Initial samples show reduced bacterial diversity and significantly different bacterial composition

Samples designated as Day Initial (DI) represent leafy greens collected after washing in a processing facility and short post-processing storage, including during shipment to Ithaca, NY. Across all replicates of the 23 DI samples tested, 254 bacterial groups were identified, representing 253 taxa, as well as a single group that included all Unclassified ASVs. The five bacterial groups with the highest average relative abundance were the genera *Pseudomonas*, *Pantoea*, *Psychrobacter*, *Exiguobacterium*, and *Bacillus* (Table 1). A mixed effects model identified 114 and 7 groups, respectively, that had significantly higher relative abundance in Harvest and DI samples. Of the 114 groups with high relative abundance in the Harvest samples, 11 had a relative abundance >1% (Table 2). The three genera with the highest log_2_ fold decrease in abundance between Harvest and DI samples included *Sphingomonas, Aerococcus,* and *Buchnera* (Table 2). The seven groups with higher abundance in DI samples included three genera with an average relative abundance of >1% in DI samples (*Pseudomonas, Erwinia*, and *Psychrobacter*) (Table 2).

**TABLE 2:** Differentially abundant bacterial and fungal groups with >1% average relative abundance in Harvest or Day Initial samples^*a*^

Groups^*a*^	Relative abundance and range (%)^*b*^ at		Log_2_ fold change^*c*^	
	Harvest	Day Initial		Significance level^*d*^
Bacteria				
Groups that are significantly enriched in Harvest samples				
*Sphingomonas*	1.8 (0.0–8.8)	0.06 (0.0–0.5)	−4.7	***
*Aerococcus*	1.7 (0.0–21.2)	0.09 (0.0–1.9)	−4.4	***
*Buchnera*	9.4 (0.0–87.5)	1.0 (0.0–15.3)	−3.4	***
*Allorhizobium-Neorhizobium-Pararhizobium-Rhizobium*	5.0 (0.0–47.0)	0.3 (0.0–3.1)	−3.3	***
*Paracoccus*	2.2 (0.0–15.3)	0.4 (0.0–7.4)	−2.9	***
*Bacillus*	11.4 (0.03–35.4)	2.7 (0.0–26.4)	−2.9	***
Unclassified ASVs	13.0 (0.3–46.7)	2.4 (0.1–32.5)	−2.8	***
*Stenotrophomonas*	1.2 (0.0–7.4)	0.2 (0.0–1.0)	−2.5	***
*Planomicrobium*	2.9 (0.0–17.1)	0.6 (0.0–4.8)	−2.4	***
*Planococcus*	2.2 (0.0–20.5)	0.7 (0.0–8.1)	−2.0	***
*Massilia*	1.4 (0.0–7.5)	1.2 (0.0–16.5)	−1.6	**
Groups that are significantly enriched in Day Initial samples				
*Pseudomonas*	8.6 (1.5–27.1)	61.4 (1.1–97.6)	2.8	***
*Erwinia*	0.4 (0.0–3.9)	2.2 (0.0–10.4)	2.3	***
*Psychrobacter*	1.1 (0.0–16.5)	4.2 (0.0–16.5)	2.0	***
Fungi				
Groups that are significantly enriched in Harvest samples				
*Epicoccum*	2.6 (0.0–7.5)	1.7 (0.03–14.4)	−0.9	*
*Curvularia*	1.8 (0.0–31.3)	0.6 (0.0–7.9)	−1.9	***
Unclassified OTUs	9.3 (0.0–54.1)	1.3 (0.0–6.5)	−2.6	***
*Fusarium*	1.4 (0.0–9.6)	0.2 (0.0–1.1)	−2.9	***
Groups that are significantly enriched in Day Initial samples				
*Cladosporium*	27.9 (7.1–57.8)	36.0 (9.4–60.9)	0.4	**
Unclassified *Pleosporales*	5.4 (0.7–16.1)	7.1 (1.1–16.3)	0.4	*
*Vishniacozyma*	4.8 (0.3–21.8)	13.5 (0.1–79.1)	1.5	***
*Cryptococcus*	0.3 (0.0–3.7)	1.5 (0.0–16.6)	3.2	***

^*a*^ Only Salinas, CA and Yuma, AZ area samples were included in this analysis, for a total of 20 Harvest and 22 DI samples; Southeast USA samples (Florida [FL] and Georgia [GA]) were not included in the data here.

^*b*^ Rarefied read counts were used to calculate relative abundance, and the relative abundance was used as the input data for this analysis. The following settings were used in the MaAsLin2 code for this analysis: analysis_method = CPLM, min_prevalence = 0, min_abundance = 0. This analysis included samples solely from the Salinas, CA area and Yuma, AZ area. This table includes taxonomic groups that (i) were detected at >1% relative abundance in either Harvest or Day Initial samples and (ii) showed significant differential abundance (*P* < 0.05). Average relative abundance is provided for each time point. The numbers in parentheses represent the range of the relative abundance of that group at that particular time point, from the minimum to the maximum value.

^*c*^ Log_2_ fold change was calculated using the model coefficient, which is part of the MaAsLin2 output. The coefficient, which is in natural log, was exponentiated and then log2 transformed [the R code was Log2 Fold Change = log2(exp(coef))]. Average relative abundance is provided as a reference but was calculated directly from the same data used as MaAsLin2 input, not MaAsLin2 output. All reported taxonomic groups had a *P*-value of <0.001 for the significance level, which was derived from the false discovery rate.

^*d*^ *P < 0.05; **P < 0.01; ***P < 0.001. Significance level was derived from the false discovery rate.

**Fig 3:**
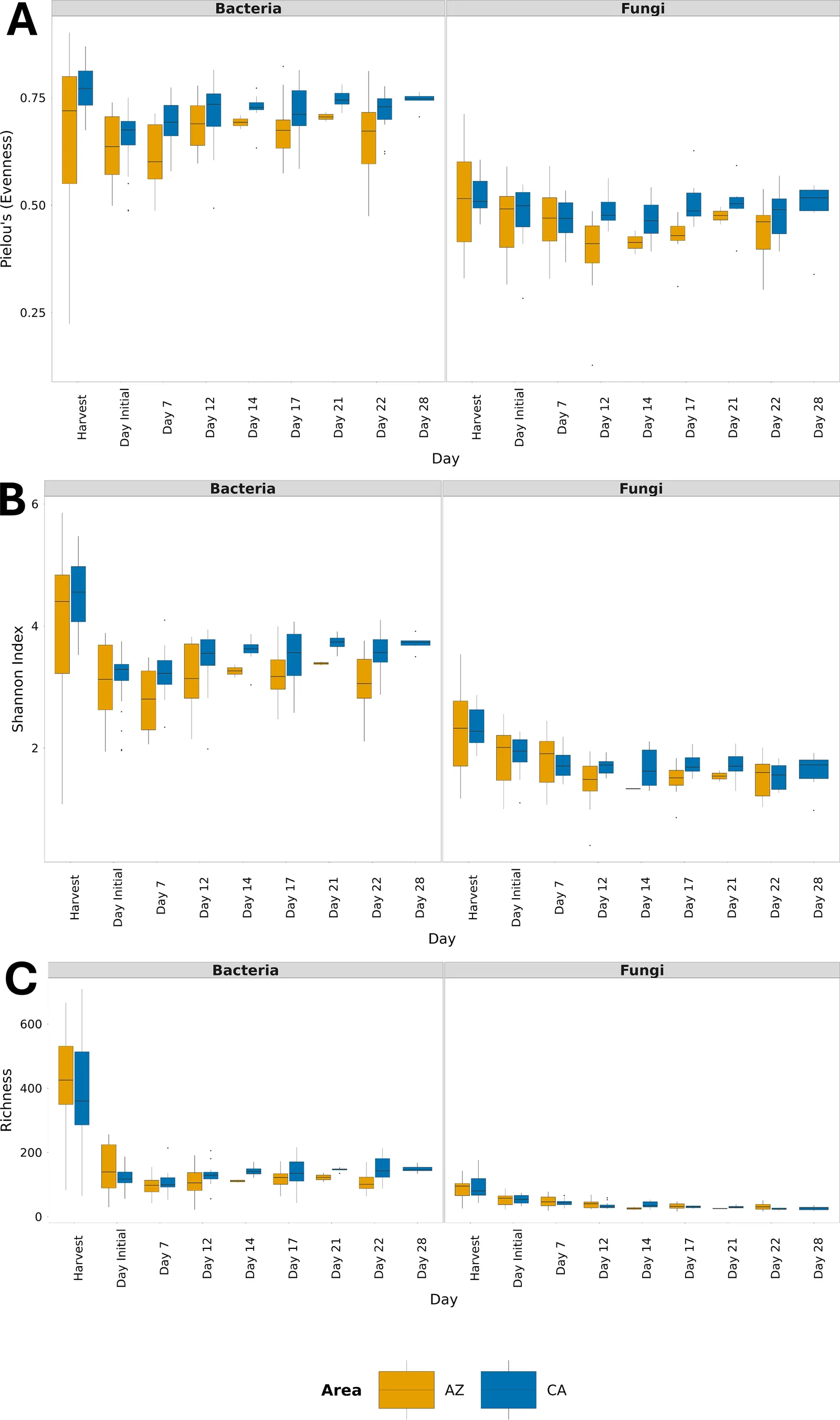
Pielou’s (Evenness), Shannon, and Richness index values for bacterial and fungal communities on baby spinach at different supply chain stages (Harvest to Day 22/28). (**A**) Pielou’s (Evenness). (**B**) Shannon Index. (**C**) Richness. Two different colors denote the area where baby spinach samples were grown (yellow denotes “AZ,” the Yuma, AZ area, and blue denotes “CA,” the Salinas, CA area).

NMDS indicates possible differences by day, as DI samples appear to assemble with one another, and Harvest samples appear to assemble with one another (Fig. 2B). PERMANOVA supported that bacterial composition was significantly (*P* = 0.001, *R*^2^ = 0.05) associated with the interaction of area (Salinas, CA vs Yuma, AZ areas) and day (i.e., Harvest vs DI samples; Table S6); however, PERMANOVA solely for DI samples revealed that area was not significant on DI (*P* > 0.05). Bacterial alpha diversity was significantly higher for Harvest samples compared to DI samples for all three indices (i.e., for Richness, Pielou’s Evenness, and Shannon) (*P* < 0.001; Fig. 3; Table S7).

Within NMDS, the two replicates of the Harvest sample from Southeast USA (SE USA) (specifically, Florida [FL]) did not overlap with the Salinas, CA area or Yuma, AZ area samples, indicating differences in bacterial composition between samples from these areas (Fig. 2A). Analysis of combined DI and Harvest samples (Fig. 2B) showed that the two FL Harvest samples assembled with the two FL DI samples.

### *Pseudomonas* dominates over shelf life with >50% relative abundance, with differences in diversity and composition driven by less abundant groups

Data for bacterial diversity over shelf life represent two slightly different data collection approaches, including (i) 18 “5-day interval” packaged samples (tested on days 7, 12, 17, and 22 [D7, D12, etc.]) and (ii) 6 “7-day interval” packaged samples (tested on days 7, 14, 21, and 28). Across both sets and all shelf-life dates (excluding the two SE USA samples), we identified 294 bacterial groups representing 293 taxa, as well as a single group that included all Unclassified ASVs; *Pseudomonas*, *Pantoea*, and *Shewanella* were among the five most common groups at both D7 and D21/22 (Table 1).

A mixed effects model was used to analyze Yuma, AZ and Salinas, CA area packaged sample sets combined by treating day of shelf life as a continuous variable; these analyses included the effects of area and day of shelf life. Three and five groups showed significantly higher relative abundance in packaged samples sourced from the Salinas, CA area and Yuma, AZ area, respectively (Table S8). A total of 62 groups showed significant changes in relative abundance over shelf life, including 21 and 41 groups, where relative abundance increased and decreased, respectively. Among the 20 most common groups across shelf life (based on average relative abundance across all days of shelf life, including DI; see Table 3), (i) 3 did not show significant changes in relative abundance over shelf life (e.g., *Pseudomonas*), (ii) 10 showed a decrease in relative abundance, and (iii) 7 showed an increase in relative abundance (Table 3). The three genera with the greatest fold decrease in relative abundance over shelf life were *Buchnera, Bacillus,* and *Planococcus*, while the three genera with the greatest fold increase were *Sphingobacterium, Flavobacterium*, and *Stenotrophomonas*. Another genus with a significant increase is *Shewanella* (Table 3), which is also one of the five most common groups at D7 and D21/22 (Table 1); in a previous culture-based study with the same samples (20), *Shewanella* was also found to be enriched in D22 samples.

**TABLE 3:** Top 20 bacterial and fungal groups (based on combined data for Salinas, CA and Yuma, AZ areas) from Day Initial to Day 21/22

Groups	Average relative abundance and range (%)^*a*^			Log_2_ fold change^*b*^	Significance level^*c*^
	All shelf-life days	At DI	At D21/D22		
Bacteria					
*Pseudomonas*	67.4 (1.1–97.8)	61.4 (1.1–97.6)	66.2 (41.7–95.5)	−0.007	−
*Pantoea*	7.0 (0.0–86.4)	13.7 (0.0–86.4)	3.9 (0.0–36.3)	−0.8	***
*Flavobacterium*	6.1 (0.0–37.0)	0.4 (0.0–3.9)	10.3 (0.0–37.0)	1.6	***
*Shewanella*	3.6 (0.0–34.1)	0.3 (0.0–4.6)	4.3 (0.0–30.1)	0.8	***
*Erwinia*	2.2 (0.0–41.2)	2.2 (0.0–10.4)	2.3 (0.2–10.7)	−0.04	−
*Psychrobacter*	1.7 (0.0–58.2)	3.5 (0.0–31.0)	0.6 (0.0–2.1)	−2.0	***
*Exiguobacterium*	1.6 (0.0–28.1)	4.2 (0.07–28.1)	0.1 (0.0–7.9)	−1.2	***
*Janthinobacterium*	1.5 (0.0–8.6)	0.3 (0.0–2.7)	2.0 (0.0–7.7)	0.9	***
*Sphingobacterium*	1.3 (0.0–31.9)	0.07 (0.0–0.9)	2.3 (0.0–14.5)	2.0	***
*Duganella*	1.1 (0.0–18.3)	0.7 (0.0–18.3)	1.6 (0.0–16.0)	0.7	***
Unclassified ASVs	0.8 (0.0–32.5)	2.4 (0.07–32.5)	0.3 (0.0–2.2)	−0.9	***
*Stenotrophomonas*	0.7 (0.0–7.8)	0.2 (0.0–1.0)	2.2 (0.0–32.5)	1.5	***
*Chryseobacterium*	0.7 (0.0–18.3)	0.2 (0.0–4.7)	1.4 (0.0–17.5)	1.2	***
*Bacillus*	0.7 (0.0–26.4)	2.7 (0.0–26.4)	0.0 (0.0–0.2)	−3.9	***
*Massilia*	0.5 (0.0–16.5)	1.2 (0.0–16.5)	0.1 (0.0–1.1)	−0.7	***
*Carnobacterium*	0.3 (0.0–18.6)	0.7 (0.0–12.9)	0.05 (0.0–1.1)	−0.9	***
*Planococcus*	0.2 (0.0–8.1)	0.7 (0.0–8.1)	0.02 (0.0–0.3)	−2.3	***
*Planomicrobium*	0.2 (0.0–4.8)	0.6 (0.0–4.8)	0.03 (0.0–0.7)	−2.0	***
*Buchnera*	0.2 (0.0–15.3)	1.0 (0.0–15.3)	0.0 (0.0–0.0)	−4.4	***
*Paenibacillus*	0.2 (0.0–4.5)	0.4 (0.0–4.5)	0.2 (0.0–1.0)	0.2	−
Fungi					
*Cladosporium*	32.7 (1.0–62.2)	36.0 (9.4–60.9)	33.6 (1.0–60.1)	−0.1	−
*Vishniacozyma*	22.0 (0.1–83.0)	13.5 (0.1–79.1)	23.7 (0.4–67.2)	0.4	***
*Alternaria*	18.1 (0.6–93.9)	20.9 (1.7–74.8)	16.8 (0.8–57.8)	−0.2	**
Unclassified *Pleosporales*	8.0 (0.0–22.7)	7.1 (1.1–16.3)	9.8 (0.0–22.7)	0.1	*
*Stemphylium*	6.8 (0.0–31.5)	7.1 (0.3–26.0)	6.7 (0.3–30.1)	−0.1	−
*Cryptococcus*	3.8 (0.0–71.4)	1.5 (0.0–16.6)	5.7 (0.0–71.4)	0.4	**
*Botrytis*	1.9 (0.0–69.1)	1.5 (0.0–12.1)	0.4 (0.0–5.5)	−0.5	*
*Sporobolomyces*	1.7 (0.0–46.7)	3.0 (0.0–46.7)	1.0 (0.0–8.1)	−0.7	***
*Epicoccum*	0.7 (0.0–14.4)	1.7 (0.03–14.4)	0.2 (0.0–0.9)	−1.2	***
Unclassified OTUs	0.6 (0.0–9.0)	1.3 (0.0–6.5)	0.3 (0.0–2.8)	−1.0	***
*Aureobasidium*	0.4 (0.0–68.6)	0.6 (0.0–4.9)	0.2 (0.0–2.5)	−0.8	***
*Candida*	0.3 (0.0–8.8)	0.6 (0.0–8.8)	0.2 (0.0–4.9)	−1.0	***
*Curvularia*	0.3 (0.0–7.9)	0.6 (0.0–7.9)	0.07 (0.0–0.6)	−1.4	***
*Papiliotrema*	0.3 (0.0–3.5)	0.2 (0.0–2.6)	0.1 (0.0–1.2)	0.1	−
*Bipolaris*	0.2 (0.0–11.8)	0.5 (0.0–8.0)	0.01 (0.0–0.1)	−1.5	***
Unclassified *Hypocreales*	0.2 (0.0–4.2)	0.04 (0.0–0.4)	0.4 (0.0–4.2)	0.6	**
*Cystofilobasidium*	0.2 (0.0–3.9)	0.04 (0.0–0.9)	0.3 (0.0–3.4)	0.3	−
Unclassified *Pleosporaceae*	0.1 (0.0–4.7)	0.3 (0.0–3.0)	0.01 (0.0–0.1)	−1.6	***
*Penicillium*	0.1 (0.0–2.4)	0.1 (0.0–0.6)	0.1 (0.0–2.4)	−0.5	**
*Fusarium*	0.1 (0.0–10.0)	0.2 (0.0–1.1)	0.04 (0.0–0.5)	−0.9	***

^*a*^ Rarefied read counts were used to calculate relative abundance, and the relative abundance was used as the input data for this analysis. The following settings were used in the MaAsLin2 code for this analysis: analysis_method = CPLM, min_prevalence = 0, and min_abundance = 0. Average relative abundance is provided for each time point. The numbers in parentheses represent the range of the relative abundance of that group at that particular time point, from the minimum to the maximum value.

^*b*^ Log_2_ fold change was calculated using the model coefficient, which is part of the MaAsLin2 output. The coefficient, which is in natural log, was exponentiated and then log_2_ transformed [the R code was Log_2_ Fold Change = log2(exp(coef))]. Average relative abundance is provided as a reference but was calculated directly from the same data used as MaAsLin2 input, not MaAsLin2 output. The log_2_ fold change was calculated for all packaged samples over time, from Day Initial to Day 22/28, including DI, D7, D12, D17, and D22 or DI, D7, D14, D21, and D28.

^*c*^ *P < 0.05; **P < 0.01; and ***P < 0.001. “–” indicates no evidence for significant differential abundance. Significance level was derived from the false discovery rate.

NMDS of the 18 “5-day interval” bacterial packaged samples (Fig. 2C) suggested possible differences between days of shelf life, as supported by a Pairwise PERMANOVA, which found that ASV composition differed significantly (*P* < 0.05, *R*^2^ = 0.08) for every combination of days (e.g., DI and D7, DI and D12), except for D17 and D22. PERMANOVA using these same samples to assess differences by area was also significant (*P* < 0.05, *R*^2^ = 0.1); however, the interaction of area and days of shelf life was not significant (*P* > 0.05; Table S6). Similar results were observed for the six “7-day interval” packaged samples; ASV composition differed significantly (*P* < 0.05, *R*^2^ = 0.1) between every combination of days, except that (i) D14 did not differ significantly (*P* > 0.05) from D21 and D28, and (ii) D21 did not differ significantly from D28. PERMANOVA for area was significant (*P* = 0.005), but the interaction of area and days of shelf life was not significant for this sample set (*P* > 0.05; Table S6).

A mixed effects model showed that 65 and 116 ASVs, respectively, significantly decreased and increased in relative abundance over shelf life (*P* < 0.05). Among the 868 *Pseudomonas* ASVs identified across all packaged samples, 23 and 39 ASVs, respectively, showed significant decreases and increases in relative abundance over shelf life; *Allorhizobium-Neorhizobium-Pararhizobium-Rhizobium* was the only other group that had both ASVs of increasing and ASVs of decreasing differential abundance. Among the packaged samples, 22 and 4 ASVs had a significantly higher relative abundance in the Salinas, CA and Yuma, AZ areas, respectively (Table S9), including 12 and 3 ASVs that were classified as *Pseudomonas* and showed higher relative abundance in the Salinas, CA and Yuma, AZ area samples, respectively (Table S9).

None of the alpha diversity indices, calculated across shelf-life days for the “5-day interval” packaged samples, differed significantly between any of the later shelf-life days (i.e., D12, D17, and D22; see Fig. 3 and Table S10). However, the values for D7 Pielou’s Evenness and Shannon indices were significantly lower than the respective values for D12, D17, and D22. Alpha diversity indices across shelf life for the “7-day interval” packaged samples also did not differ significantly between any of the later shelf-life days (i.e., D12, D17, and D22; Fig. 3 and Table S10). However, values for DI (for Pielou’s Evenness and Shannon) and DI and D7 (for Richness) were significantly lower compared to the respective values for D12, D17, and D22. Both PERMANOVA and alpha diversity index results thus suggest that significant community changes only occur earlier in shelf life.

### Mock sample results confirm that the methods used can imperfectly recover fungal diversity

The internal transcribed spacer (ITS) mock community (10 genera each at 10% relative abundance), loaded on each sequencing run at 1:1, 1:10, and 1:100 dilutions, had 9 out of 10 genera detected on all seven sequencing runs. *Cryptococcus, Cutaneotrichosporon,* and *Candida* were detected at higher than expected levels (~33%, ~20%, and ~20% relative abundance, respectively), *Saccharomyces* was detected at expected levels (~10% relative abundance), and *Penicillium, Aspergillus, Trichophyton, Fusarium,* and *Nakaseomyces* (formerly *Candida*) were detected at lower than expected levels (≤6.4% relative abundance across all sequencing runs); *Malassezia* was not detected (Table S1). This may indicate a bias in the workflow, perhaps due to the primers used, which were previously reported to over- and underestimate the abundance of different taxa (21).

### Both harvest area and time from the beginning of the growing season impact fungal diversity in Harvest samples

Across all 21 Harvest samples tested, 540 fungal groups were identified, representing 539 taxa (including 454 genera and 85 higher level taxonomic units, e.g., family; see Table S2 for a full list), as well as a single group that included all Unclassified OTUs; 18 groups were present with a relative abundance of >5% in at least one of the two replicates for at least one of the 21 tested samples (Fig. 1B). Fungal groups that showed the highest average relative abundance in Harvest samples from both the Yuma, AZ and Salinas, CA areas were *Cladosporium* and *Alternaria* (Table 1; Table S3).

Analysis with a mixed effects model accounting for the impact of area and the days from the start of the growing season identified nine and two groups that were significantly impacted by area and days from the start of the growing season, respectively. Of the four groups found to have a significantly higher relative abundance in the Salinas, CA area (Table S11), only *Fusarium* and Unclassified OTUs had >1% average relative abundance in the Salinas, CA area. Of the five groups with significantly higher relative abundance in the Yuma, AZ area (Table S11), only *Curvularia* had >1% average relative abundance. Two genera (*Stachybotrys* and *Davidiellomyces*) showed a significant decrease in relative abundance between the first and the second half of the growing season.

NMDS (Fig. 2D) assembled Salinas, CA area samples separately and more tightly than Yuma, AZ area samples, further supporting differences in fungal composition between samples from these areas, as indicated by a significant PERMDISP (*P* = 0.002; see Table S6). A PERMANOVA found that OTUs were significantly different between areas (*P* = 0.001, *R*^2^ = 0.3; Fig. 2D; Table S6).

None of the three fungal diversity metrics differed significantly between samples from the Salinas, CA area and Yuma, AZ area (Fig. 3; Table S7). Across the Harvest samples from the Salinas, CA and Yuma, AZ areas, a Spearman correlation indicated a negative correlation (*ρ* = −0.53, *P* < 0.05) between the days from the start of the growing season and Richness, indicating a decline in the number of fungal OTUs as the growing season progresses (Table S7).

### Day Initial samples show significantly reduced fungal diversity and significantly different composition at the OTU level compared to Harvest samples

The groups with the highest average relative abundance in the DI samples from the Salinas, CA area and Yuma, AZ area were *Cladosporium*, *Alternaria*, *Vishniacozyma*, Unclassified *Pleosporales*, and *Stemphylium* (Table 1). Across all replicates of the 23 DI samples tested, 299 fungal groups were identified, representing 298 taxa, as well as a single group that included all Unclassified OTUs. Analysis of group abundance data for Harvest and DI samples with a mixed effects model identified four groups, each with a significantly higher relative abundance for Harvest (e.g., *Fusarium*) and DI samples (e.g., *Cladosporium*) (Table 2).

NMDS showed that both Harvest and DI samples from the Salinas, CA area appear to assemble separately from those in the Yuma, AZ area (Fig. 2E). A PERMANOVA stratified by sample found that fungal composition (based on OTUs) was significantly different between the interaction of area (Salinas, CA vs Yuma, AZ areas) and “processing” (Harvest vs DI samples; *P* = 0.001, Table S6); however, PERMANOVA solely for DI samples revealed that area was not significant for DI (*P* = 0.1). All three alpha diversity indices were significantly higher for Harvest samples compared to DI samples (*P* < 0.01 for all three indices; Fig. 3; Table S7).

Both replicates of the FL Harvest sample did not overlap with either Salinas, CA area or Yuma, AZ area samples, indicating differences in bacterial composition between samples from these areas (Fig. 2D). Consistent with the NMDS for Harvest samples across growing areas, the FL Harvest sample replicates did not overlap with the other Harvest or DI samples in the analysis (Fig. 2E). However, FL DI samples did overlap with the Salinas, CA and Yuma, AZ area DI samples.

### Fungal diversity and composition show limited significant changes over shelf life compared to bacterial diversity and composition

Across all shelf-life dates (excluding SE USA samples), we identified 392 fungal groups, representing 391 taxa, as well as a single group that included all Unclassified OTUs; *Cladosporium*, *Vishniacozyma*, *Alternaria*, Unclassified *Pleosporales*, and *Stemphylium* represented the five most common groups for both DI and D21/22 (Table 1). A mixed effects model was used to analyze Salinas, CA and Yuma, AZ area packaged sample sets, treating day of shelf life as a continuous variable. In total, 29 groups showed significant differences in relative abundance between areas, including 9 and 20 groups with significantly higher relative abundance in the Salinas, CA and Yuma, AZ areas, respectively (Table 12). A total of 69 groups showed significant changes in relative abundance over shelf life, including 6 and 63 groups with increased and decreased relative abundance over shelf life, respectively. Among the 20 most common groups across shelf life (defined based on average relative abundance across all days of shelf life, including DI), (i) 4 did not show significant changes in relative abundance over shelf life (e.g., *Cladosporium*), (ii) 12 showed a significant decrease in relative abundance (e.g., *Alternaria*), and (iii) 4 (e.g., *Vishniacozyma*) showed a significant increase in relative abundance (Table 3). The groups that showed the greatest decrease in relative abundance were Unclassified *Pleosporaceae, Bipolaris,* and *Curvularia*, while the groups that showed the greatest increase in relative abundance were Unclassified *Hypocreales, Cryptococcus,* and *Vishniacozyma* (Table 3). For the top 20 fungal groups with significant changes in relative abundance over the course of shelf life, the log_2_ fold change range was much smaller (−1.6 to 0.6, Table 3) than the log_2_ fold change range of the top 20 bacterial groups (−4.4 to 2.0, Table 3).

NMDS values of the 18 “5-day interval” fungal data for packaged samples (Fig. 2F) suggested possible differences between days of shelf life, which was statistically supported by the pairwise PERMANOVA, which found that OTU composition differed significantly between DI and all other days over shelf life (*P* < 0.05, *R*^2^ = 0.05), and between D7 and D17 (Fig. 2F). Using these same samples, PERMANOVA for area was significant (*P* = 0.005, *R*^2^ = 0.2), and the interaction of area and days of shelf life was not significant (*P* > 0.05; Table S6). Pairwise PERMANOVA of the six “7-day interval” packaged sample data revealed that OTU composition differed significantly (*P* < 0.05, *R*^2^ = 0.1) between (i) DI and D7, (ii) DI and D28, (iii) D14 and D28, and (iv) D21 and D28. PERMANOVA was also significant (*P* = 0.005, *R*^2^ = 0.1) for area but not interaction of area and days of shelf life (*P* > 0.05; Table S6).

A mixed effects model showed that OTUs were differentially abundant by shelf-life day and by area (Table S13); 76 and 10 OTUs, respectively, decreased and increased (*P*-value < 0.05) in differential abundance over shelf life. Among 35 *Alternaria* OTUs, 1 and 3 OTUs, respectively, significantly increased and decreased in relative abundance over shelf life. Among the packaged samples, 12 and 18 OTUs had significantly higher relative abundance in the Salinas, CA and the Yuma, AZ areas, respectively. Out of 35 *Alternaria* OTUs, 1 and 3 showed significantly higher relative abundance in the Salinas, CA and Yuma, AZ areas, respectively. While typically only one or two OTUs in a given group showed overrepresentation in a given area, all three OTUs in *Stemphylium* (a genus in the top five genera in terms of relative abundance throughout shelf life; Table 1) were overrepresented in the Salinas, CA area (Table S13).

Analysis of alpha diversity indices across shelf-life days for the “5-day interval” packaged samples found no significant difference (*P* > 0.05) in Shannon and Richness indices between any of the later shelf-life days (i.e., D12, D17, and D22; see Fig. 3 and Table S10). However, the DI Shannon index was significantly lower, and the DI Richness index was significantly higher compared to the respective values for D12, D17, and D22 (see Fig. 3; Table S10). This suggests that DI samples have a wider variety of OTUs compared to samples later in shelf life, while also having a less even distribution of OTUs compared to later shelf-life days (as also supported by the lower Pielou’s Evenness values for DI and D7 compared to Days 12, 17, and 21; see Fig. 3 and Table S10). Analysis of alpha diversity indices across shelf life for the “7-day interval” packaged samples found no significant difference throughout shelf life for Shannon and Pielou’s Evenness indices. However, the DI Richness index was significantly higher compared to the values for D12, D17, and D22. Both PERMANOVA and alpha diversity index comparison results thus suggest that any significant changes in the fungal community composition occur toward the beginning of shelf life.

## DISCUSSION

In this study, we assessed the relative abundance of the bacterial and fungal communities on baby spinach using (i) 21 Harvest samples and (ii) 24 packaged sample sets, which included 23 DI samples (collected after commercial washing) and 90 packaged samples representing different shelf-life time points. Using differential abundance, ordination, and alpha diversity analyses, we showed that (i) select bacterial and fungal groups show differences between harvest areas, suggesting that the harvest area context impacts microbial diversity, (ii) commercial washing alters the spinach microbial community, more so the bacterial rather than the fungal community, (iii) *Pseudomonas* dominates the bacterial community throughout shelf life, and (iv) fungal communities in baby spinach show less changes over shelf life compared to the bacterial community. Overall, our analysis at both the genus and ASV/OTU level provides detailed insights into microbial community dynamics on spinach from harvest through the end of shelf life, which can provide a starting point for improving produce shelf life and reducing microbially mediated spoilage and defects.

### Select bacterial and fungal groups show differences between growing areas, suggesting that factors captured with a composite variable of “growing area” impact microbial diversity and that amplicon sequencing data could be used to help identify the area of origin of leafy greens

Overall, our data suggest that the relative abundance of different bacterial and fungal groups, as well as bacterial and fungal diversity, differ between growing areas. These differences were detected in both Harvest and packaged samples, suggesting that growing area factors may impact microbial communities throughout shelf life. However, the majority of the groups that showed significantly different relative abundance by area were of low relative abundance, with more common groups (e.g., *Pseudomonas* and *Bacillus*) not showing any significant differences between harvest areas. Interestingly, for bacteria and fungi, respectively, a number of reads were categorized as Unclassified ASVs and Unclassified OTUs. With these categories being particularly frequent in Harvest samples (e.g., >12% of bacterial ASVs in Harvest samples were categorized as “Unclassified ASVs”), a possible explanation is that the databases used show incomplete coverage of taxa found in agricultural environments and raw agricultural commodities. Our observations are also consistent with previous 16S rRNA and ITS amplicon sequencing studies of microbial communities on baby spinach, which similarly reported unclassified taxa (12, 15, 16), including proportions of unclassified bacterial taxa as high as 29% at the genus level in romaine lettuce leaves (15).

The common bacterial and fungal genera identified here by amplicon sequencing are similar to the most common genera previously identified on leafy greens. A previous culture-based study on the same samples (20) identified *Pseudomonas, Pantoea, Erwinia,* and *Bacillus* as common genera, generally consistent with our findings that *Bacillus*, *Pseudomonas*, *Buchnera,* and *Pantoea* were the four most common genera in Harvest samples. Frequent identification of *Buchnera* in the study reported here illustrates the value of culture-independent methods, as organisms in this genus may not grow well on standard media and with standard incubation temperatures. A number of other culture-dependent and independent studies across the world also identified *Bacillus*, *Pseudomonas*, and *Pantoea* as taxa frequently detected in the phyllosphere before washing or as common soil-dwelling organisms (12–14, 22). For fungi, our data suggest that members of the *Pleosporales* order (including the genera *Alternaria* and *Stemphylium*) dominate the fungal community at harvest across growing areas. Similarly, other studies found genera in the *Pleosporales* order (*Alternaria* and *Stemphylium*) as well as *Cladosporium* in lettuce grown in soil (14) and baby spinach grown in soil and hydroponically (13). Overall, the limited available data on the mycobiome of leafy greens suggest that both the leafy green mycobiome and bacterial microbiome in different locations (i) share many of the genera present at higher relative abundances and (ii) are characterized by the presence of a substantial number of different taxa.

While 13 and 20 bacterial groups were enriched in Harvest samples from the Salinas, CA and Yuma, AZ areas, respectively, only 10 of these groups showed >1% abundance in the area where they were found more frequently. *Buchnera* was significantly more abundant among Yuma, AZ area Harvest samples compared to Salinas, CA area Harvest samples (22.8% and 0.4% relative abundance, respectively). As *Buchnera* species are insect endosymbionts (23), this suggests higher insect pressure in the Yuma, AZ area for this particular season. Similar to our findings, Rosberg et al. (12), Brandl et al. (14), and Kgoale et al. (24) also identified *Buchnera* in the microbiome of different leafy greens collected in Sweden, California, USA, and South Africa, respectively. We also found that growing area significantly impacted select bacterial and fungal groups across packaged samples, suggesting the differences in microbial populations attributed to harvest area can persist after washing and cold storage. However, only eight bacterial groups differed in their abundance across shelf life by area. While none of the three bacterial genera that were overrepresented in Salinas, CA area packaged samples were found to be overrepresented in Salinas, CA area Harvest samples, all five bacterial genera (i.e., *Romboutsia*, *Ammoniphilus*, and *Clostridium sensu stricto* 1, 8, and 10) that were overrepresented in Yuma, AZ area packaged samples were also found to be overrepresented in Yuma, AZ area Harvest samples; all five of these groups showed low relative abundance over shelf life (with a range of 0.01%–0.1%), even though they were found throughout processing and shelf life. The fact that all five groups overrepresented in the Yuma, AZ area packaged samples are spore-forming bacteria may contribute to their ability to survive processing and storage (25–27). One of the three bacterial genera overrepresented in Salinas, CA area packaged samples, *Stenotrophomonas,* has been previously shown to survive exposure to chlorinated (~100 mg/L free chlorine) wash water in a commercial tomato packing facility, suggesting that this genus may survive in commercial spinach wash water (28).

Similar to the findings for bacterial groups, only three fungal groups detected in packaged samples were both overrepresented in either area and found at an average relative abundance >1%. Specifically, *Alternaria* was overrepresented among packaged samples associated with Yuma, AZ area samples, with a relative abundance of 27.8% and 11.7% in Yuma, AZ and Salinas, CA areas, respectively. As the Salinas, CA area has a Mediterranean climate, as opposed to the desert climate of the Yuma, AZ area, a possible explanation for this finding may be that the more arid climate of Yuma allows *Alternaria* to dominate, as supported by reports that *Alternaria* spp. can survive in soil in warm, dry climates (29, 30). Genera with an average relative abundance >1% and a significantly higher relative abundance in the Salinas, CA area packaged samples were *Botrytis* and *Stemphylium,* which are genera known to excel in humid conditions (31, 32). In addition to possible implications for produce quality and shelf life, the finding that some microbial groups were both (i) unique to specific growing areas (i.e., Salinas, CA vs Yuma, AZ) and (ii) remained detectable throughout processing and shelf life is noteworthy, as these microbial taxonomic groups could possibly be leveraged to develop novel, metagenomics-based tools for identifying or verifying a product’s origin. While application of such tools could be valuable for traceback efforts, further work is needed to confirm this, including, but not limited to, data collection over multiple seasons and supply chains, verification that communities are stable over time in a given location, and development and application of more sophisticated analysis methods to predict growing areas. However, our data are consistent with a recent study (33) that suggests that 16S rRNA amplicon sequencing data could be used for geographical origin authentication of garlic.

### Processing and washing of spinach cause more pronounced shifts in the bacterial community compared to the fungal community

The comparison between Harvest and DI samples revealed a substantial change in the bacterial community as supported by the fact that (i) all DI diversity index values were significantly lower than the harvest diversity index values, and (ii) 3 and 11 groups were significantly more relatively abundant in the DI and Harvest samples, respectively. Key groups that showed reduced relative abundance in the DI samples relative to Harvest samples included *Bacillus,* Unclassified ASVs, and *Buchnera*, suggesting that these groups may be effectively reduced by processing practices, including antimicrobial washes. On the other hand, *Pseudomonas, Erwinia,* and *Psychrobacter* were found at significantly higher relative abundance in DI samples, suggesting that they are less likely to be washed off and/or inactivated during processing and washing. Several previous studies (10, 12, 22) also reported a decrease after processing in alpha diversity measurements for bacterial communities on leafy greens. Previous studies (10, 12, 13, 22) also reported that *Pseudomonas* dominates the bacterial community on baby spinach after commercial washing/packaging, consistent with our findings that *Pseudomonas* showed >60% relative abundance on DI and throughout shelf life and the well-supported role of *Pseudomonas* in produce spoilage (34). These findings suggest that optimization of produce washes to reduce *Pseudomonas* may provide an opportunity to reduce potential agents of microbial spoilage. *Erwinia* and *Psychrobacter*, the two other genera with significantly higher relative abundance at DI, were also found throughout shelf life in our study, but at <5% relative abundance and showing either no change (*Erwinia*) or a decrease in relative abundance over shelf life (*Psychrobacter*). While *Erwinia* has been well documented to contribute to produce spoilage, *Psychrobacter* has so far primarily been linked to spoilage of meat and seafood (34, 35). This suggests that further work on the contributions of *Erwinia* to produce spoilage may be valuable and should include assessing the role of these organisms in causing produce damage (e.g., in the field, shortly after processing) that can facilitate subsequent spoilage (36), as well as investigating the overlap between plant pathogen and spoilage mechanisms of action, as many *Erwinia* species are plant pathogens (37).

Similar to our findings for bacteria, Harvest and DI samples also showed substantial differences with regard to fungal communities, as supported by the fact that (i) all DI diversity index values were significantly lower than the harvest diversity index values, and (ii) Unclassified OTUs, a taxonomic unit presumably made up of less abundant, undiscovered taxa, significantly decreased after commercial washing. Among the four fungal groups that showed increased relative abundance at DI compared to harvest, two are included in the five fungal groups with the highest relative abundance at harvest (i.e., *Cladosporium* and Unclassified *Pleosporales*). The finding that two of the top five fungal groups at harvest increase in relative abundance after processing and washing is not necessarily surprising, given that fungi are known to be less susceptible than bacteria to chlorine treatments in water (38, 39), but it could also indicate that conditions for fungal growth improve over time. Our findings are consistent with other studies, including a study (13) that found three of the top five groups identified here (i.e., *Cladosporium,* Unclassified *Pleosporales,* and *Vishniacozyma*) in washed baby spinach grown in soil or hydroponic cultivation systems. Another study (39) demonstrated that *Cladosporium,* along with *Penicillium* and *Trichoderma,* survived in settled surface water samples treated with 3 mg/L of free chlorine. Combined with a review (38) that noted that disinfectants (ClO_2_, peracetic acid, etc.) differ in their efficacy against fungi, our findings suggest that if fungi are confirmed to meaningfully contribute to quality defects in produce, further work on their sanitizer sensitivity and alternate management strategies (e.g., postharvest treatments like controlled atmospheric storage/packaging) (40) may be needed.

### While *Pseudomonas* is the predominant bacterial genus present consistently throughout shelf life, changes in the relative abundance of less common bacterial groups may provide insights into bacterial spoilage of leafy greens

Our findings that the genus *Pseudomonas* was dominant throughout shelf life on baby spinach are consistent with previous studies (10, 12, 16, 22) that reported the predominance of *Pseudomonas* on leafy greens over shelf life, as well as the well-documented role of *Pseudomonas* as an important contributor to spoilage of produce and other foods (34, 36). Interestingly, ASV-level data revealed that certain *Pseudomonas* taxa significantly increased in relative abundance throughout shelf life, while some significantly decreased in relative abundance. Brandl et al. (14) also noted that while the overall relative abundance of *Pseudomonas* increased after processing, specific *Pseudomonas* species’ relative abundance decreased after processing. Overall, more detailed identification and characterization of *Pseudomonas* spp. that grow at refrigeration temperatures and cause spoilage of leafy greens may provide opportunities to (i) reduce spoilage of these products (e.g., through validation of produce washes with regard to their ability to effectively reduce *Pseudomonas* spp. of interest) and to (ii) develop accurate shelf life and spoilage prediction models, which could be based on initial produce spoilage models (41), as well as similar models that have been developed for fluid milk spoilage (42, 43).

We also found that a number of the relatively less abundant taxonomic groups increased in relative abundance over the course of shelf life. For example, the average relative abundance of *Flavobacterium* increased significantly over shelf life (with 10.3% at D21/22 and ≤3% at DI and D7), with 32 *Flavobacterium* ASVs showing significant increases in differential abundance over shelf life. These findings are consistent with previous studies (12, 22), which reported *Flavobacterium* spp. at a higher relative abundance on leafy greens after storage. Previous studies also support that *Flavobacterium* spp. may have characteristics that would facilitate growth on plants at refrigeration temperatures. For example, *Flavobacterium* has been reported to include at least 16 documented psychrophilic or psychrotolerant species (44), and terrestrial *Flavobacterium* have been reported to encode glycoside hydrolases that target rhamnogalacturonan, a polysaccharide found in plant cell walls (45).

Despite reports of *Psychrobacter* and *Exiguobacterium* as psychrotolerant (46), the average relative abundance of these two genera significantly decreased over shelf life in our study. A previous culture-based study on the same samples (20) found that *Exiguobacterium* and *Psychrobacter* isolates made up 68 of the 2,186 isolates collected from H, D7, and D22/28, further supporting a relatively low overall abundance of these organisms. However, given that both genera were previously reported to include species that may contribute to food spoilage (35, 47), additional research may be needed to further assess their possible role in produce spoilage. Similar considerations may apply to other groups with a lower relative abundance over shelf life, as relative abundance does not necessarily correlate with an impact on spoilage (e.g., low-abundance taxa may still have a large impact on spoilage).

### The baby spinach fungal community shows fewer changes over shelf life compared to the bacterial community

Our data indicate that the baby spinach fungal community is less impacted by storage over shelf life, compared to the bacterial community, as supported by (i) a smaller log_2_ fold change range in differentially abundant groups over shelf life, (ii) fewer significant differences in the three alpha diversity indices over shelf life, and (iii) the observation that four of the top five genera are consistent from harvest throughout shelf life. Our data also suggest that the fungal community over shelf life is primarily driven by a few taxa, including *Cladosporium,* which was the most abundant fungal genus from harvest until Day 21/22. The fact that we identified only two *Cladosporium* OTUs in the whole data set furthermore suggests that there are likely few, yet dominant *Cladosporium* taxa, which might make management of this genus simpler (although this finding may also be due to the lower discriminatory power of ITS amplicon sequencing). A previous culture-independent study of the phyllosphere of lettuce grown in Salinas, CA identified two specific *Cladosporium* species (*Cladosporium cladosporiodes* and *Cladosporium phlei*), with the genus *Cladosporium* apparently found in all samples and showing up to >15% relative abundance in at least one sample (14). Overall, our data provide one of the few baselines on fungal diversity in leafy greens throughout production, processing, and refrigerated storage, providing potentially valuable groundwork for future studies and hypothesis generation on the contributions of fungi to produce quality and spoilage. However, it is important to take into account the limits of amplicon sequencing approaches used, which include limited standardization of ITS methods compared to 16S rRNA methods, which may lead to increased bias (as also supported by our findings with fungal mock community control) and reduced ability to compare findings between studies. Other well-documented limitations, which apply to both fungi and bacteria, include that amplicon sequencing (i) measures relative abundance, not absolute abundance (48), (ii) does not provide any data on microbial activity (49), and (iii) has limited discriminatory power (50).

### Conclusion

Our study supports that bacterial and fungal communities on baby spinach are influenced by both (i) preharvest factors (i.e., growing region and seasonality) and (ii) postharvest factors (e.g., commercial washing/packaging and storage over shelf life), but with fungal communities less impacted by these factors compared to bacterial communities. While growing regions and seasonality may have some impact on leafy green microbiomes, these factors appear to predominantly impact less abundant taxonomic groups. On the other hand, while produce processing and particularly washes clearly have differential impacts on produce microbiomes, with some spoilage genera (e.g., *Pseudomonas*) becoming predominant after produce washing, community changes over refrigerated storage suggest that only specific taxa within some genera (e.g., *Pseudomonas*) may be major contributors to spoilage. Consequently, the characterization of *Pseudomonas* species and strains that can cause spoilage, as well as the development and validation of interventions targeting these *Pseudomonas,* may be valuable. Furthermore, a comprehensive understanding of microbial drivers of spoilage and the associated mechanisms that promulgate spoilage (e.g., enzymatic degradation) will be important and could be achieved through the use of additional -omics technologies (e.g., shotgun metagenomics, metabolomics, and transcriptomics). These data could yield targeted approaches to reduce produce spoilage, e.g., by targeting specific spoilage microbiota or mechanisms (e.g., similar enzymatic activities found across different organisms).

## MATERIALS AND METHODS

### Sample collection

This project involved 16S rRNA gene and ITS amplicon sequencing-based characterization of fresh baby spinach samples obtained between December 2021 and December 2022 to assess the relative abundance of the bacterial and fungal communities on baby spinach (i) after harvest, (ii) after washing, and (iii) throughout shelf life. Collection of the baby spinach samples and culture-dependent analyses of the samples were previously described (20). Briefly, the timeline of the study (i.e., December 2021–December 2022) was selected as it allowed for the characterization of the spinach microbiome across the major growing regions and seasons in the United States, including production in (i) the Salinas, CA area (April–November) and (ii) the Yuma, AZ area and the Imperial Valley desert area in California (November–April) (6–8). Samples tested typically represented two samplings per month from a supply chain that sourced spinach mainly from California and Arizona, but also occasionally from the SE USA (Florida and Georgia). The bi-monthly sampling frequency was selected based on the logistical capability of the research team and industry collaborator; thus, the sampling frequency and resulting sample size represent a convenience-based sampling strategy and sample size. During each sampling, we aimed to collect samples from the same order (i.e., lot) (i) after harvest (“Harvest samples”) and (ii) after commercial washing and packaging (“Packaged” samples). Even though the growing regions differed, all lots were processed in the same facility; processing consistently included a chlorine wash. Harvest and packaged samples were selected as they were broadly representative of two industry-relevant “states” of fresh baby spinach, including: (i) “raw” product (represented by the Harvest samples) and (ii) finished product (represented by the packaged samples).

Ultimately, we conducted 27 samplings of baby spinach between December 2021 and December 2022, with details provided in our previous publication (20). These 27 samplings are identified by IDs that comprised the month and year of sampling, e.g., 0122_A denotes the first (A) sampling in January 2022, while 0122_B represents the second (B) sampling in January 2022. The 27 samplings represent spinach that was grown in (i) Salinas Valley, Monterey County, CA (referred to as “Salinas, CA area,” *n* = 15); (ii) Yuma, AZ (*n* = 9) and Imperial Valley, CA (*n* = 1) (referred to as “Yuma, AZ area,” as these two growing areas are in the same geographic region); and (iii) Florida (FL) (*n* = 1), and Georgia (GA) (*n* = 1) (referred to as SE USA), see Tables S14 and S15). Across these 27 samplings, we collected (i) 23 Harvest samples and (ii) 25 packaged samples; these sample numbers are not equal to the number of samplings (i.e., 27 samplings) since, during some samplings, we were unable to collect either the (i) Harvest sample (i.e., for samplings 0122_A, 0822_B, 1122_A, and 1122_B) or (ii) packaged sample (i.e., for samplings 0722_B and 1222_C). Of the 27 samplings, we collected Harvest and packaged samples from the same lot (i.e., order) in 17 samplings; for one of these 17 samplings, one Harvest sample (i.e., 1222_B) was not sequenced, resulting in 16 samplings of Harvest and packaged samples that were obtained from the same lot. In the remaining 10 of 27 samplings, we either (i) collected Harvest and packaged samples within 14 days of each other or (ii) only collected Harvest or packaged samples (as detailed above). Of the 23 Harvest and 25 packaged samples, select samples were not included in the final data set used for analysis, including (i) the 1222_B Harvest samples, which were not sequenced (as mentioned above); (ii) the 0722_B Harvest samples, which were excluded due to a delay in transit of Harvest samples from the grower to Ithaca, NY; and (iii) the 0922_B packaged samples, which were excluded from shelf-life analysis due to lack of metadata about the area of cultivation. SE USA Harvest and packaged samples were not included in certain analyses due to the small sample size and lack of a Harvest sample from GA (see Tables S14 and S15). Thus, the final data set used for analysis included 21 Harvest samples (12 from the Salinas, CA area, 8 from the Yuma, AZ area, and 1 from FL, SE USA) and 24 packaged samples (13 from the Salinas, CA area, 9 from the Yuma, AZ area, and 2 from SE USA).

### Sample preparation and testing

All samples were shipped on ice and tested within 6 h of receipt in Ithaca, NY. More specifically, Harvest samples were only tested upon receipt (i.e., a single time point of testing). Harvest samples were shipped by the grower to Ithaca, NY, on the day of harvest or the day after harvest. Packaged samples were tested upon receipt to obtain data for the Day Initial time point and then also portioned into 12 units of 25 g each, which were stored in separate Whirl-Pak bags at 4°C for up to 28 days to characterize microbial populations over shelf life. The day of shelf life when DI testing occurred varied by sampling, from 1 to 5 days post-packaging, depending on shipping logistics (for additional details, see https://github.com/FSL-MQIP/2022_spinach_sampling_counts_isolates/blob/main/counts/data/raw/day_initial_shelf_life.csv). The packaged samples, which were stored as 25-g portions, were subsequently tested either on (i) days 7 (D7), 12 (D12), 17 (D17), and 22 (D22) post-packaging (“5-day interval,” which includes eight Yuma, AZ area samples, eight Salinas, CA area samples, and two SE USA samples) or (ii) on days 7, 14 (D14), 21 (D21), and 28 (D28) post-packaging (“7-day interval,” which includes one Yuma, AZ area sample and five Salinas, CA area samples). While our initial design called for testing at 5-day intervals, some samples were erroneously tested at a 7-day interval; these data, however, could be analyzed as some testing days were common to both sampling schemes and as days in shelf life were analyzed as a continuous variable. Additionally, due to an incubator malfunction, we were unable to obtain data at the following time points: (i) D28 for 0422_A, (ii) D21 and D28 for 0422_B, and (iii) D14, D21, and D28 for 0522_A. One packaged sample (1122_B) could not be tested on DI due to shipping delays.

For each sample (e.g., a given Harvest sample and a given D22 sample), three replicates were collected and used for each (i) plating (as detailed in reference 20) and (ii) collection of cell pellets for amplicon sequencing. Three sample replicates of each baby spinach sample were processed by fully immersing three 25-g portions of spinach leaves in Butterfield’s phosphate buffer (BPB, 1×), followed by stomaching to obtain spinach homogenates, as previously described (20). After stomaching, two 10 mL aliquots of each stomached sample were pipetted into two separate 40 mL sterile centrifuge tubes, followed by centrifugation at 10,000*g* for 15 min at 4°C. After decanting the supernatant, each pellet was resuspended in 1 mL of ultrapure water. Each resuspended pellet was transferred to a separate 2 mL microcentrifuge tube, followed by centrifugation at 10,000*g* for 15 min at room temperature. Subsequently, 1 mL of the supernatant was removed from each tube, and the pellet was resuspended in any remaining liquid. For each sample processing event, a negative control (NC) of BPB was processed in the same manner as spinach homogenates, producing one NC sample paired with the three sample replicates processed at each time point. This process resulted in duplicate pellets for each of the three sample replicates and for each associated NC sample; pellets were stored at −20°C until DNA extraction.

### Selection of samples for DNA extraction and amplicon sequencing

Of the three replicates for each sample, we selected two replicates for DNA extraction and amplicon sequencing. For Harvest samples, the two sample replicates were selected based on previously reported aerobic plate count data; specifically, replicates were selected to represent the replicate with the median bacterial count and a second randomly selected replicate. For DI-D22/D28, the two replicates were selected using the same criteria (median bacterial count and a second randomly selected replicate), but based on psychrotolerant plate counts (20).

### DNA extraction and PCR

DNA extraction was carried out on one of the two pellets collected for each sample replicate, as described above. The exception to this was sample 16S_0522_A_H_3_CA, where DNA extracted from the first pellet yielded low read counts during sequencing. The remaining pellet for this replicate was then used for DNA extraction and sequencing. DNA was extracted from thawed pellets using the Qiagen DNeasy PowerSoil Pro Kit (Qiagen, Germantown, MD, USA) according to the manufacturer’s instructions. Extracted DNA was quantified with the Qubit High Sensitivity dsDNA Quantification Assay Kits (Invitrogen, Waltham, MA, USA), and nucleic acid purity was assessed with a Nanodrop 2000c Spectrophotometer (Thermo Fisher Scientific, Waltham, MA, USA). DNA was stored at −20°C until PCR was performed.

Duplicate 16S rRNA gene amplicon pools per sample replicate were generated with 335f/769r primers (51) using GoTaq Flexi DNA Polymerase (Promega Corp, Madison, WI, USA) and the following thermocycling method: 95°C for 3 min; 30 cycles of 95°C for 30 s, 55°C for 30 s, and 72°C for 35 s; 72°C for 10 min; with a hold at 4°C. Duplicate ITS amplicon pools per sample replicate were generated with 5.8S-Fun/ITS4-Fun primers (21) using GoTaq Flexi DNA Polymerase (Promega) and the following touchdown thermocycling parameters: 95°C for 2 min; 10 cycles of 95°C for 30 s, 58°C decreasing by 0.5°C each cycle to 53°C for 30 s, and 72°C for 35 s; 72°C for 10 min; 20 cycles of 95°C for 30 s, 35°C for 30 s, and 72°C for 35 s; 72°C for 10 min; with a hold at 4°C. For both the 16S rRNA gene and the ITS amplicons, agarose gel electrophoresis was used to confirm PCR amplification for each individual PCR reaction. Duplicate PCR reactions were pooled into one pool for sequencing.

### Mock community samples

The ZymoBIOMICS Microbial Community DNA Standard II (Log Distribution) (Zymo Research, Irvine, CA, USA) diluted to a concentration of 1 ng/µL was used as a positive control for 16S rRNA gene sample replicates (17). The ATCC Mycobiome Genomic DNA Mix (diluted to concentrations of 1, 0.1, and 0.01 ng/µL) (ATCC, Manassas, VA, USA) was used as a positive control for ITS sample replicates.

### Sequencing

Pooled amplicons were sent to the Biotechnology Resource Center Genomics Facility (RRID: SCR_021727) at the Cornell Institute of Biotechnology for library preparation and sequencing on an Illumina MiSeq using a v2 500 bp kit with 2 × 250 Paired Ends (Illumina, San Diego, CA, USA). 16S rRNA gene and ITS amplicons generated from each DNA sample were sequenced together to increase the complexity during sequencing. Each sequencing run also included (i) a mock community, which was used as a positive control, and (ii) two randomly selected sample processing NCs (DNA extracted from BPB processed in the same manner as the samples). Samples that had read counts below the highest read count of all NC samples were re-sequenced.

### Bioinformatic analyses

Processing of 16S rRNA gene reads was performed in R v.4.2.1 using DADA2 (52). Primers were removed from 16S rRNA gene forward and reverse reads via CutAdapt (53). Then, reads were filtered and trimmed with the filterAndTrim function, using the following parameters: truncLen = c(230, 230), maxN = 0, maxEE = c(2, 2), truncQ = 2, minLen = 50, rm.phix = TRUE. Paired reads were merged, and chimeras were removed. Taxonomic classification of 16S rRNA gene ASVs was performed with the SILVA v.138.1 database (54).

ITS gene reads were processed using the naïve Bayes classifier in the QIIME2 pipeline (55). Primer removal was performed separately on forward and reverse reads, using Trimmomatic v.0.39 (56); forward and reverse reads were subsequently merged with BBmerge (57). Merged reads were processed and filtered using the naïve Bayes classifier QIIME2 pipeline (55) in QIIME 2023.9, clustering reads with a 97% similarity. Following chimera removal, homopolymers greater than eight bases in length were removed with the RESCRIPt plugin (58). Taxonomic classification was performed using the UNITE fungal database v.10.0 (59). The taxonomy and OTU tables were then imported into R, with the qiime2R package, for further data analysis and visualization.

Contaminant removal for both 16S rRNA gene and ITS reads was performed using the decontam package (60) using the “combined method” and a threshold of 0.05; the mock community samples were excluded from the sample set when decontam was run. For the 16S rRNA gene and ITS data, 46 ASVs and 28 OTUs, respectively, were identified and removed from the samples. We also removed (i) ASVs and OTUs with <2 reads per sample, and (ii) ASVs and OTUs classified as “Chloroplast,” “mitochondria,” or “Viridiplantae” (the latter for ITS reads only). Read counts were rarified with rarefy_even_depth for further analyses.

### Statistical analysis

Statistical analyses and data visualization were completed in R v.4.2.1 using the vegan, tidyverse, phyloseq, maditr, pairwiseAdonis, theseus, dunn.test, FSA, rcompanion, emmeans, and ggplot2 packages. MaAsLin2 (18) was used to create mixed effects models to calculate differential abundance using both relative abundance (for genus/taxonomic group level analysis) and read counts per ASV/OTU (for ASV/OTU level analysis). Within MaAsLin2, “CPLM” was used as the analysis method (with no log transformation). A combination of the Month and Lot (i.e., 0122_A, January A) was used as the random effect; area, time from the start of the season, and shelf-life date were used as fixed effects for different analyses. For analyses performed at the genus level, the calculated relative abundance was used as an input, with the minimum prevalence and minimum abundance filtering standards set to 0. For analyses performed at the ASV/OTU level, the read count was used as an input, with the minimum prevalence and minimum abundance filtering standards set to 0.1 and 0.01, respectively. Differential abundance by time from the start of the growing season and day of shelf life were analyzed as continuous variables in MaAsLin2.

The permutation design for PERMANOVA was specified using the “how” function (from the permute package) to ensure the sample replicates were not permuted against each other, but rather permuted as a unit (61). To run a PERMANOVA with the “how” function, the number of samples for each level of the factors being tested had to be equivalent in size; thus, samples were randomly selected to ensure each level of a factor would have an equivalent sample size in the analysis. All nonmetric multidimensional scaling was performed with Bray-Curtis values. *t*-tests were used to test for differences between alpha diversity indices between two groups (e.g., Yuma, AZ area and Salinas, CA area); we elected not to use a Bonferroni correction because only three *t*-tests (one for each of the three alpha diversity indices) were performed (a corrected *P*-value cutoff would have been 0.013).

The start of the growing season was defined as (i) 12 February 2022 for the Salinas, CA area 2022 season, (ii) 8 September 2021 for the Yuma, AZ area 2021–2022 season, and (iii) 7 September 2022 for the Yuma, AZ area 2022–2023 season; these dates were obtained from the collaborating growers. For our purposes, the growing season starts when planting in a particular area starts. Based on this information, we separated the growing season by area into (i) first half (April–mid-July for Salinas, CA area and December–mid-February for Yuma, AZ area) and (ii) second half (mid-July–November for Salinas, CA area and mid-February–April for Yuma, AZ area).

## Data Availability

Sequences are available on NCBI under Sequence Read Archive number PRJNA1219179. Code for the subsequent analyses is available on GitHub (https://github.com/FSL-MQIP/Baby-Spinach-Leaf-Microbiome).

## SUPPLEMENTAL MATERIAL

Supplemental Material spectrum.04007-25-s0001.docx Tables S1 to S15.

ASM does not own the copyrights to Supplemental Material that may be linked to, or accessed through, an article. The authors have granted ASM a non-exclusive, world-wide license to publish the Supplemental Material files. Please contact the corresponding author directly for reuse.
