# Supplementary material for: Commercial washing and storage over shelf life impact bacterial communities more than the fungal communities on baby spinach
Source: Microbiol Spectr. 2026 Jun 15;14(7):e04007-25. doi: 10.1128/spectrum.04007-25 (PMC13340335; doi:10.1128/spectrum.04007-25)
Supplement: Supplemental Material — Tables S1 to S15. [file spectrum.04007-25-s0001.docx]

Supplementary Table 1. Bacterial and Fungal Mock Community Results

|  | **Defined Composition (%)** | **Relative Abundance (%) from Sequencing Run** | | | | | | |
| --- | --- | --- | --- | --- | --- | --- | --- | --- |
| **Taxonomic Groups** |  | **1** | **2** | **3** | **4** | **5** | **6** | **7** |
| ***Bacteria*** |  |  |  |  |  |  |  |  |
| *Listeria* | 89.1 | 92.6 | 93.5 | 92.2 | 93.0 | 93.4 | 89.8 | 94.1 |
| *Pseudomonas* | 8.9 | 6.9 | 5.9 | 7.0 | 6.4 | 5.8 | 9.3 | 5.4 |
| *Bacillus* | 0.9 | 0.1 | 0.3 | 0.4 | 0.4 | 0.4 | 0.5 | 0.1 |
| *Escherichia-Shigella* | 0.09 | 0.1 | 0.1 | 0.1 | 0.1 | 0.1 | 0.2 | 0.0 |
| *Salmonella* | 0.09 | 0.1 | 0.1 | 0.1 | 0.1 | 0.1 | 0.1 | 0.0 |
| *Lactobacillus* | 0.009 | 0.0 | 0.0 | 0.0 | 0.0 | 0.0 | 0.0 | 0.0 |
| *Enterococcus* | 0.0009 | 0.0 | 0.0 | 0.0 | 0.0 | 0.0 | 0.0 | 0.0 |
| *Staphylococcus* | 0.00009 | 0.0 | 0.0 | 0.0 | 0.0 | 0.0 | 0.0 | 0.0 |
| ***Fungi: 1:100 Dilution*** | | |  |  |  |  |  |  |
| *Aspergillus* | 10.0 | 3.3 | 2.9 | 3.9 | 1.8 | 3.1 | 4.3 | 3.2 |
| *Candida* | 10.0 | 22.0 | 18.2 | 19.8 | 26.8 | 20.2 | 22.9 | 20.7 |
| *Cryptococcus* | 10.0 | 22.5 | 25.8 | 20.2 | 18.5 | 21.4 | 18.5 | 18.5 |
| *Cutaneotrichosporon* | 10.0 | 32.5 | 30.1 | 30.5 | 37.9 | 32.1 | 32.8 | 34.1 |
| *Fusarium* | 10.0 | 0.05 | 0.05 | 0.15 | 0.04 | 0.01 | 0.03 | 0.01 |
| *Malassezia* | 10.0 | 0.0 | 0.0 | 0.0 | 0.0 | 0.0 | 0.0 | 0.0 |
| *Nakaseomyces* | 10.0 | 2.7 | 4.3 | 3.8 | 1.2 | 2.9 | 2.4 | 3.1 |
| *Penicillium* | 10.0 | 4.9 | 2.6 | 4.7 | 2.8 | 5.4 | 6.4 | 5.9 |
| *Saccharomyces* | 10.0 | 11.1 | 14.9 | 15.7 | 10.5 | 13.6 | 11.2 | 13.3 |
| *Trichophyton* | 10.0 | 0.8 | 1.1 | 1.2 | 0.5 | 1.4 | 1.3 | 1.2 |

Supplementary Table 2. Taxonomic classification of bacterial and fungal groups identified in Harvest samples

| **Bacterial Groups** | **Taxonomic Level from SILVA 16S rRNA database^1^** | **Fungal Groups** | **Taxonomic Level from UNITE fungal database^2^** |
| --- | --- | --- | --- |
| 1174-901-12 | N/A^3^ | *Acanthobasidium* | Genus |
| AKYG587 | N/A^3^ | *Acarospora* | Genus |
| *Abditibacterium* | Genus | *Achaetomium* | Genus |
| *Abiotrophia* | Genus | *Acremonium* | Genus |
| *Acanthopleuribacter* | Genus | *Acrostalagmus* | Genus |
| *Acaricomes* | Genus | *Actinomucor* | Genus |
| *Acetitomaculum* | Genus | *Aculeastrum* | Genus |
| *Acetoanaerobium* | Genus | *Aeminium* | Genus |
| *Achromobacter* | Genus | *Agaricus* | Genus |
| *Acidibacter* | Genus | *Agrocybe* | Genus |
| *Acidiphilium* | Genus | *Albatrellus* | Genus |
| *Acidothermus* | Genus | *Albicollum* | Genus |
| *Acidovorax* | Genus | *Albifimbria* | Genus |
| *Acinetobacter* | Genus | *Allocanariomyces* | Genus |
| *Acrocarpospora* | Genus | *Alnicola* | Genus |
| *Actinocorallia* | Genus | *Alternaria* | Genus |
| *Actinomadura* | Genus | *Amphinema* | Genus |
| *Actinoplanes* | Genus | *Amphobotrys* | Genus |
| *Adhaeribacter* | Genus | *Amyloxenasma* | Genus |
| *Advenella* | Genus | *Annulohypoxylon* | Genus |
| *Aerococcus* | Genus | *Antarctolichenia* | Genus |
| *Aeromicrobium* | Genus | *Aphelidium* | Genus |
| *Aeromonas* | Genus | *Apiorhynchostoma* | Genus |
| *Aerosphaera* | Genus | *Aposphaeria* | Genus |
| *Aetherobacter* | Genus | *Arachnomyces* | Genus |
| *Afifella* | Genus | *Arrhenia* | Genus |
| *Afipia* | Genus | *Arthrinium* | Genus |
| *Agathobacter* | Genus | *Arxotrichum* | Genus |
| *Aggregicoccus* | Genus | *Ascotricha* | Genus |
| *Agromyces* | Genus | *Aspergillus* | Genus |
| *Ahniella* | Genus | *Athelia* | Genus |
| *Alcaligenes* | Genus | *Atractidochium* | Genus |
| *Algoriella* | Genus | *Aureobasidium* | Genus |
| *Algoriphagus* | Genus | *Auxarthron* | Genus |
| *Alicyclobacillus* | Genus | *Bacillicladium* | Genus |
| *Aliicoccus* | Genus | *Baeospora* | Genus |
| *Aliifodinibius* | Genus | *Begerowomyces* | Genus |
| *Aliihoeflea* | Genus | *Berkeleyomyces* | Genus |
| *Alishewanella* | Genus | *Betamyces* | Genus |
| *Alistipes* | Genus | *Bezerromyces* | Genus |
| *Alkalibacter* | Genus | *Biatoropsis* | Genus |
| *Alkalibacterium* | Genus | *Bipolaris* | Genus |
| *Allorhizobium-Neorhizobium*  *-Pararhizobium-Rhizobium* | Genus | *Biscogniauxia* | Genus |
| *Allostreptomyces* | Genus | *Bisifusarium* | Genus |
| *Alsobacter* | Genus | *Bjerkandera* | Genus |
| *Altererythrobacter* | Genus | *Blumeria* | Genus |
| *Amaricoccus* | Genus | *Bolbitius* | Genus |
| *Aminobacter* | Genus | *Botryobasidium* | Genus |
| *Ammoniibacillus* | Genus | *Botryotrichum* | Genus |
| *Ammoniphilus* | Genus | *Botrytis* | Genus |
| *Anaerobacillus* | Genus | *Bovista* | Genus |
| *Anaerobacterium* | Genus | *Brunneofusispora* | Genus |
| *Anaerocolumna* | Genus | *Brunneomyces* | Genus |
| *Anaeromyxobacter* | Genus | *Buckleyzyma* | Genus |
| *Anaerosolibacter* | Genus | *Buellia* | Genus |
| *Anaerosporobacter* | Genus | *Bulleribasidium* | Genus |
| *Aneurinibacillus* | Genus | *Burgoa* | Genus |
| *Antarcticibacterium* | Genus | *Butlerelfia* | Genus |
| *Apibacter* | Genus | *Byssomerulius* | Genus |
| *Aquamicrobium* | Genus | *Calvatia* | Genus |
| *Aquamonas* | Genus | *Camarosporium* | Genus |
| *Aquibacillus* | Genus | *Camillea* | Genus |
| *Aquicella* | Genus | *Canariomyces* | Genus |
| *Arboricoccus* | Genus | *Candelaria* | Genus |
| *Archangium* | Genus | *Candida* | Genus |
| *Arcobacter* | Genus | *Capnobotryella* | Genus |
| *Arcticibacter* | Genus | *Catenulomyces* | Genus |
| *Arenibacter* | Genus | *Celosporium* | Genus |
| *Arenimonas* | Genus | *Cephaliophora* | Genus |
| *Arsenicitalea* | Genus | *Cephalotrichiella* | Genus |
| *Arthrobacter* | Genus | *Cephalotrichum* | Genus |
| *Atlantibacter* | Genus | *Cercospora* | Genus |
| *Atopococcus* | Genus | *Chaetocapnodium* | Genus |
| *Atopostipes* | Genus | *Chaetomium* | Genus |
| *Aureibacillus* | Genus | *Chaetosphaeronema* | Genus |
| *Aureimonas* | Genus | *Chalara* | Genus |
| *Azoarcus* | Genus | *Chondrostereum* | Genus |
| *Azohydromonas* | Genus | *Chrysosporium* | Genus |
| *Azospirillum* | Genus | *Chrysothrix* | Genus |
| *Azotobacter* | Genus | *Cinereomyces* | Genus |
| BIyi10 | N/A^3^ | *Cippumomyces* | Genus |
| BRH-c57 | N/A^3^ | *Citeromyces* | Genus |
| *Bacillus* | Genus | *Cladophialophora* | Genus |
| *Bauldia* | Genus | *Cladosporium* | Genus |
| *Bdellovibrio* | Genus | *Clarireedia* | Genus |
| *Belnapia* | Genus | *Clavispora* | Genus |
| *Bhargavaea* | Genus | *Clitopilus* | Genus |
| *Bifidobacterium* | Genus | *Colacogloea* | Genus |
| *Blastococcus* | Genus | *Comoclathris* | Genus |
| *Blastomonas* | Genus | *Coniocessia* | Genus |
| *Blautia* | Genus | *Coniochaeta* | Genus |
| *Bordetella* | Genus | *Coniophora* | Genus |
| *Bosea* | Genus | *Coniosporium* | Genus |
| *Brachybacterium* | Genus | *Coniothyrium* | Genus |
| *Branchiibius* | Genus | *Conocybe* | Genus |
| *Brevibacillus* | Genus | *Constantinomyces* | Genus |
| *Brevundimonas* | Genus | *Coprinellus* | Genus |
| *Brochothrix* | Genus | *Coprinopsis* | Genus |
| *Brucella* | Genus | *Coprinus* | Genus |
| *Bryobacter* | Genus | *Cortinarius* | Genus |
| *Buchnera* | Genus | *Cosmospora* | Genus |
| *Burkholderia-Caballeronia-Paraburkholderia* | Genus | *Crassisporium* | Genus |
| *Buttiauxella* | Genus | *Crustomyces* | Genus |
| C1-B045 | N/A^3^ | *Cryptococcus* | Genus |
| CL500-29 marine group | N/A^3^ | *Curvibasidium* | Genus |
| *Caedibacter* | Genus | *Curvularia* | Genus |
| *Caenimonas* | Genus | *Cutaneotrichosporon* | Genus |
| *Caenispirillum* | Genus | *Cyclaneusma* | Genus |
| *Caldalkalibacillus* | Genus | *Cyphellophora* | Genus |
| *Caldicoprobacter* | Genus | *Cystobasidium* | Genus |
| *Candidatus* Alysiosphaera | Genus | *Cystofilobasidium* | Genus |
| *Candidatus* Entotheonella | Genus | *Cystotheca* | Genus |
| *Candidatus* Hemobacterium | Genus | *Cytidia* | Genus |
| *Candidatus* Lariskella | Genus | *Cytospora* | Genus |
| *Candidatus* Megaira | Genus | *Dacrymyces* | Genus |
| *Candidatus* Methylopumilus | Genus | *Dactylella* | Genus |
| *Candidatus* Ovatusbacter | Genus | *Daedaleopsis* | Genus |
| *Candidatus* Paracaedibacter | Genus | *Daldinia* | Genus |
| *Candidatus* Phytoplasma | Genus | *Davidiellomyces* | Genus |
| *Candidatus* Planktoluna | Genus | *Dematiopleospora* | Genus |
| *Candidatus* Regiella | Genus | *Diatrype* | Genus |
| *Candidatus* Schneideria | Genus | *Dichlaena* | Genus |
| *Candidatus* Soleaferrea | Genus | *Didymella* | Genus |
| *Candidatus* Solibacter | Genus | *Dioszegia* | Genus |
| *Candidatus* Symbiobacter | Genus | *Diploicia* | Genus |
| *Candidatus* Thiobios | Genus | *Dipodascus* | Genus |
| *Candidimonas* | Genus | *Dissoconium* | Genus |
| *Carnobacterium* | Genus | *Diutina* | Genus |
| *Caryophanon* | Genus | *Donadinia* | Genus |
| *Catellatospora* | Genus | *Dothiorella* | Genus |
| *Catenisphaera* | Genus | *Dothistroma* | Genus |
| *Caulobacter* | Genus | *Eichleriella* | Genus |
| *Cedecea* | Genus | *Elasticomyces* | Genus |
| *Cellulomonas* | Genus | *Elmerina* | Genus |
| *Cellulosilyticum* | Genus | *Enterocarpus* | Genus |
| *Cellvibrio* | Genus | *Entoloma* | Genus |
| *Cereibacter* | Genus | *Epicoccum* | Genus |
| *Cesiribacter* | Genus | *Erysiphe* | Genus |
| *Chelativorans* | Genus | *Erythrobasidium* | Genus |
| *Chiayiivirga* | Genus | *Evernia* | Genus |
| *Chishuiella* | Genus | *Exidia* | Genus |
| *Chitinibacter* | Genus | *Exobasidium* | Genus |
| *Chitinophaga* | Genus | *Exophiala* | Genus |
| *Christensenellaceae R-7 group* | N/A^3^ | *Exserohilum* | Genus |
| *Chryseobacterium* | Genus | *Fagicola* | Genus |
| *Chryseoglobus* | Genus | *Fibroporia* | Genus |
| *Chryseolinea* | Genus | *Fibulobasidium* | Genus |
| *Chryseomicrobium* | Genus | *Filobasidium* | Genus |
| *Chthonobacter* | Genus | *Flavodon* | Genus |
| *Chujaibacter* | Genus | *Fomes* | Genus |
| *Chungangia* | Genus | *Fomitella* | Genus |
| *Citrifermentans* | Genus | *Fomitiporella* | Genus |
| *Citrobacter* | Genus | *Fusarium* | Genus |
| *Clostridioides* | Genus | *Geastrum* | Genus |
| *Clostridium* | Genus | *Gelidatrema* | Genus |
| *Clostridium sensu stricto 1* | Genus | *Genolevuria* | Genus |
| *Clostridium sensu stricto 10* | Genus | *Geomyces* | Genus |
| *Clostridium sensu stricto 12* | Genus | *Geosmithia* | Genus |
| *Clostridium sensu stricto 13* | Genus | *Geotrichum* | Genus |
| *Clostridium sensu stricto 3* | Genus | *Gerhardtia* | Genus |
| *Clostridium sensu stricto 5* | Genus | *Gibberella* | Genus |
| *Clostridium sensu stricto 6* | Genus | *Glarea* | Genus |
| *Clostridium sensu stricto 7* | Genus | *Gloeophyllum* | Genus |
| *Clostridium sensu stricto 8* | Genus | *Golovinomyces* | Genus |
| *Clostridium sensu stricto 9* | Genus | *Graphiola* | Genus |
| *Cohnella* | Genus | *Gymnoascus* | Genus |
| *Comamonas* | Genus | *Gymnopilus* | Genus |
| *Conexibacter* | Genus | *Halobyssothecium* | Genus |
| *Constrictibacter* | Genus | *Hannaella* | Genus |
| *Corallococcus* | Genus | *Hansfordia* | Genus |
| *Corticibacterium* | Genus | *Harknessia* | Genus |
| *Couchioplanes* | Genus | *Henningsomyces* | Genus |
| *Coxiella* | Genus | *Hericium* | Genus |
| *Croceicoccus* | Genus | *Heterobasidion* | Genus |
| *Cupriavidus* | Genus | *Hongkongmyces* | Genus |
| *Curtobacterium* | Genus | *Hormodochis* | Genus |
| *Curvibacter* | Genus | *Hormonema* | Genus |
| *Cystobacter* | Genus | *Hortaea* | Genus |
| *Cytophaga* | Genus | *Hydeomyces* | Genus |
| *Dechloromonas* | Genus | *Hymenochaete* | Genus |
| *Deefgea* | Genus | *Hymenochaetopsis* | Genus |
| *Defluviicoccus* | Genus | *Hyphoderma* | Genus |
| *Defluviimonas* | Genus | *Hyphodermella* | Genus |
| *Defluviitalea* | Genus | *Hyphodontia* | Genus |
| *Delftia* | Genus | *Hypotrachyna* | Genus |
| *Desemzia* | Genus | *Hypoxylon* | Genus |
| *Desertibacter* | Genus | *Hysteropatella* | Genus |
| *Desulfallas-Sporotomaculum* | Genus | *Inopinatum* | Genus |
| *Desulfitobacterium* | Genus | *Insolibasidium* | Genus |
| *Desulfofarcimen* | Genus | *Iodophanus* | Genus |
| *Desulfohalotomaculum* | Genus | *Irpex* | Genus |
| *Desulfosporosinus* | Genus | *Jaapia* | Genus |
| *Desulfotomaculum* | Genus | *Kazachstania* | Genus |
| *Desulfovibrio* | Genus | *Keissleriella* | Genus |
| *Devosia* | Genus | *Knufia* | Genus |
| *Dietzia* | Genus | *Kwoniella* | Genus |
| *Dinghuibacter* | Genus | *Laetiporus* | Genus |
| *Domibacillus* | Genus | *Lambertella* | Genus |
| *Dongia* | Genus | *Lambiella* | Genus |
| *Duganella* | Genus | *Lapidomyces* | Genus |
| *Dyadobacter* | Genus | *Lasiobolidium* | Genus |
| E1B-B3-114 | N/A^3^ | *Lectera* | Genus |
| *Edaphobaculum* | Genus | *Lecythophora* | Genus |
| *Elizabethkingia* | Genus | *Lentinus* | Genus |
| Ellin6055 | N/A^3^ | *Lenzites* | Genus |
| Ellin6067 | N/A^3^ | *Lepista* | Genus |
| *Emticicia* | Genus | *Lepraria* | Genus |
| *Endobacter* | Genus | *Leptosphaeria* | Genus |
| *Enhydrobacter* | Genus | *Leptospora* | Genus |
| *Ensifer* | Genus | *Leucosporidium* | Genus |
| *Enterobacillus* | Genus | *Leucothecium* | Genus |
| *Enterobacter* | Genus | *Leveillula* | Genus |
| *Enterococcus* | Genus | *Libertasomyces* | Genus |
| *Epulopiscium* | Genus | *Lithohypha* | Genus |
| *Erwinia* | Genus | *Lophiostoma* | Genus |
| *Erythrobacter* | Genus | *Lophodermium* | Genus |
| *Escherichia-Shigella* | Genus | *Lycoperdon* | Genus |
| *Exiguobacterium* | Genus | *Lyomyces* | Genus |
| *Facklamia* | Genus | *Magnohelicospora* | Genus |
| *Falsibacillus* | Genus | *Malassezia* | Genus |
| *Falsirhodobacter* | Genus | *Malbranchea* | Genus |
| *Falsochrobactrum* | Genus | *Marchandiomyces* | Genus |
| *Ferritrophicum* | Genus | *Melampsora* | Genus |
| *Ferrovibrio* | Genus | *Melanocarpus* | Genus |
| *Ferruginibacter* | Genus | *Meristemomyces* | Genus |
| *Fictibacillus* | Genus | *Meruliopsis* | Genus |
| *Filibacter* | Genus | *Meruliporia* | Genus |
| *Filomicrobium* | Genus | *Metacapnodium* | Genus |
| *Flaviaesturariibacter* | Genus | *Metschnikowia* | Genus |
| *Flavisolibacter* | Genus | *Microascus* | Genus |
| *Flavitalea* | Genus | *Microcera* | Genus |
| *Flavobacterium* | Genus | *Microdochium* | Genus |
| *Fluviicola* | Genus | *Microthelia* | Genus |
| *Fontibacillus* | Genus | *Mollisia* | Genus |
| *Fonticella* | Genus | *Monosporascus* | Genus |
| *Friedmanniella* | Genus | *Montagnula* | Genus |
| *Frigoribacterium* | Genus | *Mortierella* | Genus |
| *Frisingicoccus* | Genus | *Mrakia* | Genus |
| *Fulvivirga* | Genus | *Mucor* | Genus |
| GKS98 freshwater group | N/A^3^ | *Mycena* | Genus |
| *Gaiella* | Genus | *Mycoacia* | Genus |
| *Gallionella* | Genus | *Mycocalicium* | Genus |
| *Geminicoccus* | Genus | *Mycosphaerella* | Genus |
| *Gemmatimonas* | Genus | *Myxomphalia* | Genus |
| *Gemmatirosa* | Genus | *Myxotrichum* | Genus |
| *Geobacillus* | Genus | *Naganishia* | Genus |
| *Geodermatophilus* | Genus | *Nemania* | Genus |
| *Geomicrobium* | Genus | *Neocamarosporium* | Genus |
| *Georgfuchsia* | Genus | *Neocatenulostroma* | Genus |
| *Gilliamella* | Genus | *Neocladosporium* | Genus |
| *Glutamicibacter* | Genus | *Neocucurbitaria* | Genus |
| *Glycomyces* | Genus | *Neodendryphiella* | Genus |
| *Gordonia* | Genus | *Neodevriesia* | Genus |
| *Gracilibacillus* | Genus | *Neoerysiphe* | Genus |
| *Hafnia-Obesumbacterium* | Genus | *Neooccultibambusa* | Genus |
| *Hahella* | Genus | *Neophaeococcomyces* | Genus |
| *Haliangium* | Genus | *Neophaeosphaeria* | Genus |
| *Haloactinopolyspora* | Genus | *Neophaeotheca* | Genus |
| *Halobacillus* | Genus | *Neoschizothecium* | Genus |
| *Halomonas* | Genus | *Neoscytalidium* | Genus |
| *Haloplasma* | Genus | *Neovaginatispora* | Genus |
| *Hartmannibacter* | Genus | *Neurospora* | Genus |
| HdN1 | N/A^3^ | *Niebla* | Genus |
| *Hephaestia* | Genus | *Nigrograna* | Genus |
| *Herbaspirillum* | Genus | *Nigrospora* | Genus |
| *Herbidospora* | Genus | *Nonappendiculata* | Genus |
| *Herbinix* | Genus | *Nothophaeotheca* | Genus |
| *Hydrogenophaga* | Genus | *Occultifur* | Genus |
| *Hymenobacter* | Genus | *Ochrocladosporium* | Genus |
| *Hyphomicrobium* | Genus | *Ochroconis* | Genus |
| *Iamia* | Genus | *Oedocephalum* | Genus |
| *Ideonella* | Genus | *Ophiognomonia* | Genus |
| *Ilumatobacter* | Genus | *Pachyramichloridium* | Genus |
| *Izhakiella* | Genus | *Panaeolus* | Genus |
| JCM 18997 | N/A^3^ | *Panellus* | Genus |
| JTB255 marine benthic group | N/A^3^ | *Papiliotrema* | Genus |
| *Jannaschia* | Genus | *Paraconiothyrium* | Genus |
| *Janthinobacterium* | Genus | *Paraloratospora* | Genus |
| *Jeotgalibaca* | Genus | *Paraphaeosphaeria* | Genus |
| *Jeotgalibacillus* | Genus | *Paraphoma* | Genus |
| *Jeotgalicoccus* | Genus | *Parasarocladium* | Genus |
| KCM-B-112 | N/A^3^ | *Parateratosphaeria* | Genus |
| *Kaistia* | Genus | *Parmotrema* | Genus |
| *Ketobacter* | Genus | *Penicillium* | Genus |
| *Klebsiella* | Genus | *Penidiella* | Genus |
| *Kluyvera* | Genus | *Peniophora* | Genus |
| *Knoellia* | Genus | *Peniophorella* | Genus |
| *Kocuria* | Genus | *Perenniporia* | Genus |
| *Kosakonia* | Genus | *Periconia* | Genus |
| *Koukoulia* | Genus | *Peroneutypa* | Genus |
| *Krasilnikovia* | Genus | *Pestalotiopsis* | Genus |
| *Kribbella* | Genus | *Petrophila* | Genus |
| *Kroppenstedtia* | Genus | *Peziza* | Genus |
| *Kushneria* | Genus | *Phaeoacremonium* | Genus |
| *Kytococcus* | Genus | *Phaeobotryon* | Genus |
| *Labrys* | Genus | *Phaeococcomyces* | Genus |
| *Laceyella* | Genus | *Phaeodothis* | Genus |
| *Lachnoclostridium* | Genus | *Phaeosphaeria* | Genus |
| Lachnospiraceae AC2044 group | N/A^3^ | *Phaeothecoidea* | Genus |
| Lachnospiraceae UCG-002 | N/A^3^ | *Phanerochaete* | Genus |
| Lachnospiraceae UCG-010 | N/A^3^ | *Phellinus* | Genus |
| *Lachnotalea* | Genus | *Phialemonium* | Genus |
| *Lacihabitans* | Genus | *Phialocephala* | Genus |
| *Lacticigenium* | Genus | *Phialophora* | Genus |
| *Lactococcus* | Genus | *Phlebia* | Genus |
| *Latilactobacillus* | Genus | *Phlebiopsis* | Genus |
| *Lautropia* | Genus | *Pholiota* | Genus |
| *Legionella* | Genus | *Phoma* | Genus |
| *Lelliottia* | Genus | *Phragmocamarosporium* | Genus |
| *Lentibacillus* | Genus | *Pilidium* | Genus |
| *Leptothrix* | Genus | *Piloderma* | Genus |
| *Leuconostoc* | Genus | *Pinaceicola* | Genus |
| *Ligilactobacillus* | Genus | *Pirex* | Genus |
| *Lihuaxuella* | Genus | *Piskurozyma* | Genus |
| *Limibaculum* | Genus | *Pithoascus* | Genus |
| *Limnobacter* | Genus | *Plectania* | Genus |
| *Limnohabitans* | Genus | *Pleiochaeta* | Genus |
| *Longimicrobium* | Genus | *Plicaria* | Genus |
| *Longispora* | Genus | *Pluteus* | Genus |
| *Lonsdalea* | Genus | *Podaxis* | Genus |
| *Luedemannella* | Genus | *Podosphaera* | Genus |
| *Luteimonas* | Genus | *Podospora* | Genus |
| *Luteococcus* | Genus | *Preussia* | Genus |
| *Lysinibacillus* | Genus | *Pringsheimia* | Genus |
| *Lysobacter* | Genus | *Psathyrella* | Genus |
| MN 122.2a | N/A^3^ | *Pseudocercospora* | Genus |
| MND1 | N/A^3^ | *Pseudocercosporella* | Genus |
| *Macellibacteroides* | Genus | *Pseudogymnoascus* | Genus |
| *Macrococcus* | Genus | *Pseudohormonema* | Genus |
| *Malikia* | Genus | *Pseudoplectania* | Genus |
| *Marinilactibacillus* | Genus | *Pseudosydowia* | Genus |
| *Marinobacter* | Genus | *Pseudotaeniolina* | Genus |
| *Marinomonas* | Genus | *Pseudothielavia* | Genus |
| *Marmoricola* | Genus | *Psoroglaena* | Genus |
| *Massilia* | Genus | *Puccinia* | Genus |
| *Melghirimyces* | Genus | *Punctularia* | Genus |
| *Mesorhizobium* | Genus | *Pyrenochaeta* | Genus |
| *Methylarcula* | Genus | *Pyrenophora* | Genus |
| *Methylibium* | Genus | *Pyxidiophora* | Genus |
| *Methylobacillus* | Genus | *Rachicladosporium* | Genus |
| *Methylobacterium-Methylorubrum* | Genus | *Ramalina* | Genus |
| *Methylocaldum* | Genus | *Ramimonilia* | Genus |
| *Methylocella* | Genus | *Ramularia* | Genus |
| *Methylocystis* | Genus | *Resinicium* | Genus |
| *Methylophaga* | Genus | *Resinoporia* | Genus |
| *Methylophilus* | Genus | *Resupinatus* | Genus |
| *Methylorosula* | Genus | *Rhexothecium* | Genus |
| *Methylotenera* | Genus | *Rhinocladiella* | Genus |
| *Microaerobacter* | Genus | *Rhizopus* | Genus |
| *Microbacterium* | Genus | *Rhodosporidiobolus* | Genus |
| *Microlunatus* | Genus | *Rhodotorula* | Genus |
| *Micromonospora* | Genus | *Rigidoporus* | Genus |
| *Microvirga* | Genus | *Rinodina* | Genus |
| *Mobilitalea* | Genus | *Rosellinia* | Genus |
| *Modestobacter* | Genus | *Roseograndinia* | Genus |
| *Modicisalibacter* | Genus | *Rutstroemia* | Genus |
| *Moellerella* | Genus | *Sagenomella* | Genus |
| *Moheibacter* | Genus | *Saitoella* | Genus |
| *Monoglobus* | Genus | *Saitozyma* | Genus |
| *Morganella* | Genus | *Salinomyces* | Genus |
| *Mucilaginibacter* | Genus | *Sarcinomyces* | Genus |
| *Mycetocola* | Genus | *Sarcopodium* | Genus |
| *Mycobacterium* | Genus | *Sarcotrochila* | Genus |
| *Mycoplana* | Genus | *Sarocladium* | Genus |
| *Myroides* | Genus | *Sawadaea* | Genus |
| *Myxococcus* | Genus | *Scedosporium* | Genus |
| NK4A214 group | N/A^3^ | *Schizothecium* | Genus |
| *Naasia* | Genus | *Schizothyrium* | Genus |
| *Nakamurella* | Genus | *Scolecoxyphium* | Genus |
| *Nannocystis* | Genus | *Scopulariopsis* | Genus |
| *Natranaerovirga* | Genus | *Scorias* | Genus |
| *Natronobacillus* | Genus | *Scytalidium* | Genus |
| *Naumannella* | Genus | *Seimatosporium* | Genus |
| *Neisseria* | Genus | *Selenophoma* | Genus |
| Neo-b11 | N/A^3^ | *Septobasidium* | Genus |
| *Neorhizobium* | Genus | *Septoria* | Genus |
| *Nesterenkonia* | Genus | *Sidera* | Genus |
| *Niastella* | Genus | *Sistotrema* | Genus |
| *Nibrella* | Genus | *Sistotremastrum* | Genus |
| *Nibribacter* | Genus | *Skeletocutis* | Genus |
| *Nitrobacter* | Genus | *Slooffia* | Genus |
| *Nitrococcus* | Genus | *Solicoccozyma* | Genus |
| *Nitrosococcus* | Genus | *Sorocybe* | Genus |
| *Nitrosomonas* | Genus | *Spegazzinia* | Genus |
| *Nitrosospira* | Genus | *Sphagnurus* | Genus |
| *Nitrospira* | Genus | *Spiromastigoides* | Genus |
| *Niveitalea* | Genus | *Sporobolomyces* | Genus |
| *Nocardia* | Genus | *Sporormia* | Genus |
| *Nocardioides* | Genus | *Sporormiella* | Genus |
| *Nonomuraea* | Genus | *Stachybotrys* | Genus |
| *Nordella* | Genus | *Stagonospora* | Genus |
| *Novibacillus* | Genus | *Starmerella* | Genus |
| *Noviherbaspirillum* | Genus | *Staurosphaeria* | Genus |
| *Novosphingobium* | Genus | *Steccherinum* | Genus |
| OM27 clade | N/A^3^ | *Stemphylium* | Genus |
| OM60(NOR5) clade | N/A^3^ | *Stereum* | Genus |
| *Oceanicella* | Genus | *Stictis* | Genus |
| *Oceaniovalibus* | Genus | *Subramaniula* | Genus |
| *Oceanisphaera* | Genus | *Sulcosporium* | Genus |
| *Oceanobacillus* | Genus | *Symbiotaphrina* | Genus |
| *Ochrobactrum* | Genus | *Symmetrospora* | Genus |
| *Oerskovia* | Genus | *Taeniolella* | Genus |
| *Ohtaekwangia* | Genus | *Talaromyces* | Genus |
| *Oligoflexus* | Genus | *Tamaricicola* | Genus |
| *Orbus* | Genus | *Taphrina* | Genus |
| *Oribacterium* | Genus | *Tausonia* | Genus |
| *Ornithinibacillus* | Genus | *Teretispora* | Genus |
| *Ornithinicoccus* | Genus | *Teunia* | Genus |
| *Ornithinimicrobium* | Genus | *Thelebolus* | Genus |
| *Ornithobacterium* | Genus | *Thelephora* | Genus |
| *Orrella* | Genus | *Thermomyces* | Genus |
| *Oxalicibacterium* | Genus | *Thielavia* | Genus |
| *Oxalophagus* | Genus | *Torula* | Genus |
| *Oxobacter* | Genus | *Toxicocladosporium* | Genus |
| P3OB-42 | N/A^3^ | *Trametes* | Genus |
| PMMR1 | N/A^3^ | *Trametopsis* | Genus |
| *Paenarthrobacter* | Genus | *Trechispora* | Genus |
| *Paenibacillus* | Genus | *Tremella* | Genus |
| *Paeniclostridium* | Genus | *Trichaptum* | Genus |
| *Paeniglutamicibacter* | Genus | *Tricharina* | Genus |
| *Paenisporosarcina* | Genus | *Trichoderma* | Genus |
| *Pajaroellobacter* | Genus | *Trichosporiella* | Genus |
| *Palleronia-Pseudomaribius* | Genus | *Trichosporon* | Genus |
| *Pantoea* | Genus | *Tubulicrinis* | Genus |
| *Parabacteroides* | Genus | *Tulosesus* | Genus |
| *Parablastomonas* | Genus | *Tulostoma* | Genus |
| *Paraclostridium* | Genus | *Tyromyces* | Genus |
| *Paracoccus* | Genus | *Udeniomyces* | Genus |
| *Paraherbaspirillum* | Genus | Unclassified *Acarosporaceae* | Family |
| *Parapedobacter* | Genus | Unclassified Agaricales | Order |
| *Parapusillimonas* | Genus | Unclassified Agaricomycetes | Class |
| *Parasegetibacter* | Genus | Unclassified *Amphisphaeriaceae* | Family |
| *Parasporobacterium* | Genus | Unclassified Arthoniomycetes | Class |
| *Parviterribacter* | Genus | Unclassified *Ascodesmidaceae* | Family |
| *Pasteuria* | Genus | Unclassified Ascomycota | Division |
| *Paucibacter* | Genus | Unclassified *Aspergillaceae* | Family |
| *Paucisalibacillus* | Genus | Unclassified *Atheliaceae* | Family |
| *Pectobacterium* | Genus | Unclassified Auriculariales | Order |
| *Pedobacter* | Genus | Unclassified Basidiomycota | Division |
| *Pedomicrobium* | Genus | Unclassified *Bionectriaceae* | Family |
| *Pelagibacterium* | Genus | Unclassified Boletales | Order |
| *Pelomonas* | Genus | Unclassified Botryosphaeriales | Order |
| *Peptoclostridium* | Genus | Unclassified Cantharellales | Order |
| *Peredibacter* | Genus | Unclassified Capnodiales | Order |
| *Phaselicystis* | Genus | Unclassified *Ceratostomataceae* | Family |
| *Phaseolibacter* | Genus | Unclassified *Chaetomiaceae* | Family |
| *Phenylobacterium* | Genus | Unclassified Chaetothyriales | Order |
| *Phreatobacter* | Genus | Unclassified Chytridiomycota | Division |
| *Phyllobacterium* | Genus | Unclassified *Coniothyriaceae* | Family |
| *Phytohabitans* | Genus | Unclassified *Corticiaceae* | Family |
| *Pigmentiphaga* | Genus | Unclassified *Cryptococcaceae* | Family |
| *Piscibacillus* | Genus | Unclassified Diaporthales | Order |
| *Piscinibacter* | Genus | Unclassified *Didymellaceae* | Family |
| *Planktotalea* | Genus | Unclassified Dothideales | Order |
| *Planococcus* | Genus | Unclassified Dothideomycetes | Class |
| *Planomicrobium* | Genus | Unclassified *Dothidotthiaceae* | Family |
| *Planomonospora* | Genus | Unclassified Eurotiales | Order |
| Plot4-2H12 | N/A^3^ | Unclassified Eurotiomycetes | Class |
| *Polaromonas* | Genus | Unclassified *Filobasidiaceae* | Family |
| *Polyangium* | Genus | Unclassified *Geastraceae* | Family |
| *Polycladomyces* | Genus | Unclassified *Graphostromataceae* | Family |
| *Polynucleobacter* | Genus | Unclassified *Helicobasidiaceae* | Family |
| *Pontibacillus* | Genus | Unclassified Helotiales | Order |
| *Pontibacter* | Genus | Unclassified *Herpotrichiellaceae* | Family |
| *Porphyrobacter* | Genus | Unclassified Hymenochaetales | Order |
| *Povalibacter* | Genus | Unclassified Hypocreales | Order |
| *Proteiniclasticum* | Genus | Unclassified Lecanoromycetes | Class |
| *Proteocatella* | Genus | Unclassified *Lipomycetaceae* | Family |
| *Providencia* | Genus | Unclassified *Meruliaceae* | Family |
| *Pseudaminobacter* | Genus | Unclassified Microascales | Order |
| *Pseudarcicella* | Genus | Unclassified Microstromatales | Order |
| *Pseudarthrobacter* | Genus | Unclassified *Mollisiaceae* | Family |
| *Pseudenhygromyxa* | Genus | Unclassified *Mycosphaerellaceae* | Family |
| *Pseudochrobactrum* | Genus | Unclassified Myriangiales | Order |
| *Pseudoclavibacter* | Genus | Unclassified *Neocamarosporiaceae* | Family |
| *Pseudoduganella* | Genus | Unclassified *Neodevriesiaceae* | Family |
| *Pseudofulvimonas* | Genus | Unclassified *Onygenaceae* | Family |
| *Pseudogracilibacillus* | Genus | Unclassified Onygenales | Order |
| *Pseudohongiella* | Genus | Unclassified *Orbiliaceae* | Family |
| *Pseudolabrys* | Genus | Unclassified Ostropales | Order |
| *Pseudomonas* | Genus | Unclassified OTUs^4^ | Unclassified^4^ |
| *Pseudonocardia* | Genus | Unclassified *Parmeliaceae* | Family |
| *Pseudorhizobium* | Genus | Unclassified *Pezizaceae* | Family |
| *Pseudorhodobacter* | Genus | Unclassified Pezizales | Order |
| *Pseudorhodoferax* | Genus | Unclassified Pezizomycetes | Class |
| *Pseudorhodoplanes* | Genus | Unclassified Pezizomycotina | Subdivision |
| *Pseudoxanthomonas* | Genus | Unclassified *Phaeosphaeriaceae* | Family |
| *Psychrobacillus* | Genus | Unclassified *Phanerochaetaceae* | Family |
| *Psychrobacter* | Genus | Unclassified *Plectosphaerellaceae* | Family |
| *Psychroglaciecola* | Genus | Unclassified *Pleosporaceae* | Family |
| *Puia* | Genus | Unclassified Pleosporales | Order |
| *Pullulanibacillus* | Genus | Unclassified Polyporales | Order |
| *Pusillimonas* | Genus | Unclassified *Protomycetaceae* | Family |
| *Qipengyuania* | Genus | Unclassified *Pseudeurotiaceae* | Family |
| *Rahnella* | Genus | Unclassified *Pucciniaceae* | Family |
| Rahnella1 | N/A^3^ | Unclassified Pucciniales | Order |
| *Ralstonia* | Genus | Unclassified *Pyronemataceae* | Family |
| *Ramlibacter* | Genus | Unclassified *Rhytismataceae* | Family |
| *Reyranella* | Genus | Unclassified Rozellomycota | Subkingdom |
| *Rheinheimera* | Genus | Unclassified Russulales | Order |
| *Rhizobacter* | Genus | Unclassified Saccharomycetales | Order |
| *Rhizorhapis* | Genus | Unclassified Saccharomycetes | Class |
| *Rhodanobacter* | Genus | Unclassified Sebacinales | Order |
| *Rhodococcus* | Genus | Unclassified Sordariales | Order |
| *Rhodocytophaga* | Genus | Unclassified Sordariomycetes | Class |
| *Rhodoferax* | Genus | Unclassified *Sporormiaceae* | Family |
| *Rhodoluna* | Genus | Unclassified *Stereaceae* | Family |
| *Rhodoplanes* | Genus | Unclassified *Stictidaceae* | Family |
| *Rhodothermus* | Genus | Unclassified *Teloschistaceae* | Family |
| *Rickettsia* | Genus | Unclassified Teloschistales | Order |
| *Risungbinella* | Genus | Unclassified *Teratosphaeriaceae* | Family |
| *Rivibacter* | Genus | Unclassified Trechisporales | Order |
| *Romboutsia* | Genus | Unclassified Tremellales | Order |
| *Roseisolibacter* | Genus | Unclassified Tremellomycetes | Class |
| *Rosenbergiella* | Genus | Unclassified Tubeufiales | Order |
| *Roseomonas* | Genus | Unclassified *Xylariaceae* | Family |
| *Rothia* | Genus | Unclassified Xylariales | Order |
| *Rubellimicrobium* | Genus | *Uromyces* | Genus |
| *Rubrimonas* | Genus | *Usnea* | Genus |
| *Rubrivirga* | Genus | *Uwebraunia* | Genus |
| *Rubrivivax* | Genus | *Valsa* | Genus |
| *Rubrobacter* | Genus | *Vermiconia* | Genus |
| *Rugamonas* | Genus | *Veronaea* | Genus |
| *Ruminiclostridium* | Genus | *Verrucocladosporium* | Genus |
| *Ruminococcus* | Genus | *Verticillium* | Genus |
| *Rummeliibacillus* | Genus | *Vishniacozyma* | Genus |
| SM1A02 | N/A^3^ | *Wallemia* | Genus |
| SN8 | N/A^3^ | *Waynea* | Genus |
| SWB02 | N/A^3^ | *Westerdykella* | Genus |
| SZB85 | N/A^3^ | *Whalleya* | Genus |
| *Saccharibacillus* | Genus | *Wilsonomyces* | Genus |
| *Saccharomonospora* | Genus | *Wolfiporia* | Genus |
| *Saccharopolyspora* | Genus | *Xenasma* | Genus |
| *Saccharothrix* | Genus | *Xenasmatella* | Genus |
| *Salinibacillus* | Genus | *Xenomeris* | Genus |
| *Salinicoccus* | Genus | *Xylaria* | Genus |
| *Salinicola* | Genus | *Xylobolus* | Genus |
| *Salinisphaera* | Genus | *Xylodon* | Genus |
| *Salinispora* | Genus | *Zalaria* | Genus |
| *Salipaludibacillus* | Genus | *Zopfiella* | Genus |
| *Salirhabdus* | Genus |  |  |
| *Saliterribacillus* | Genus |  |  |
| *Salmonella* | Genus |  |  |
| *Samsonia* | Genus |  |  |
| *Sandaracinus* | Genus |  |  |
| *Sandarakinorhabdus* | Genus |  |  |
| *Schumannella* | Genus |  |  |
| *Scopulibacillus* | Genus |  |  |
| *Sedimentibacter* | Genus |  |  |
| *Sediminibacillus* | Genus |  |  |
| *Sediminibacterium* | Genus |  |  |
| *Segetibacter* | Genus |  |  |
| *Seinonella* | Genus |  |  |
| *Seohaeicola* | Genus |  |  |
| *Serinicoccus* | Genus |  |  |
| *Serratia* | Genus |  |  |
| *Shewanella* | Genus |  |  |
| *Shimazuella* | Genus |  |  |
| *Shinella* | Genus |  |  |
| *Siccibacter* | Genus |  |  |
| *Simplicispira* | Genus |  |  |
| *Sinibacillus* | Genus |  |  |
| *Skermanella* | Genus |  |  |
| *Snodgrassella* | Genus |  |  |
| *Solibacillus* | Genus |  |  |
| *Solirubrobacter* | Genus |  |  |
| *Soonwooa* | Genus |  |  |
| *Sorangium* | Genus |  |  |
| *Sphaerisporangium* | Genus |  |  |
| *Sphingoaurantiacus* | Genus |  |  |
| *Sphingobacterium* | Genus |  |  |
| *Sphingobium* | Genus |  |  |
| *Sphingomonas* | Genus |  |  |
| *Sphingopyxis* | Genus |  |  |
| *Sphingorhabdus* | Genus |  |  |
| *Sphingosinicella* | Genus |  |  |
| *Spirillospora* | Genus |  |  |
| *Sporacetigenium* | Genus |  |  |
| *Sporobacter* | Genus |  |  |
| *Sporocytophaga* | Genus |  |  |
| *Sporosarcina* | Genus |  |  |
| *Stackebrandtia* | Genus |  |  |
| *Stakelama* | Genus |  |  |
| *Staphylococcus* | Genus |  |  |
| *Stenotrophomonas* | Genus |  |  |
| *Steroidobacter* | Genus |  |  |
| *Streptococcus* | Genus |  |  |
| *Streptomyces* | Genus |  |  |
| *Streptosporangium* | Genus |  |  |
| Subgroup 10 | N/A^3^ |  |  |
| *Sulfurifustis* | Genus |  |  |
| *Sulfurimonas* | Genus |  |  |
| *Sulfurisoma* | Genus |  |  |
| *Sulfuritalea* | Genus |  |  |
| *Syntrophomonas* | Genus |  |  |
| TM7a | N/A^3^ |  |  |
| *Tabrizicola* | Genus |  |  |
| *Telluria* | Genus |  |  |
| *Telmatospirillum* | Genus |  |  |
| *Tenuibacillus* | Genus |  |  |
| *Tepidamorphus* | Genus |  |  |
| *Tepidibacter* | Genus |  |  |
| *Tepidimicrobium* | Genus |  |  |
| *Terribacillus* | Genus |  |  |
| *Terrimonas* | Genus |  |  |
| *Terrisporobacter* | Genus |  |  |
| *Tersicoccus* | Genus |  |  |
| *Tessaracoccus* | Genus |  |  |
| *Thalassobacillus* | Genus |  |  |
| *Thalassobaculum* | Genus |  |  |
| *Thermincola* | Genus |  |  |
| *Thermithiobacillus* | Genus |  |  |
| *Thermoactinomyces* | Genus |  |  |
| *Thermobacillus* | Genus |  |  |
| *Thermocatellispora* | Genus |  |  |
| *Thermoflavimicrobium* | Genus |  |  |
| *Thermomonas* | Genus |  |  |
| *Thermostaphylospora* | Genus |  |  |
| *Thermotunica* | Genus |  |  |
| *Thioalkalispira-Sulfurivermis* | Genus |  |  |
| *Thiobacillus* | Genus |  |  |
| *Thiothrix* | Genus |  |  |
| *Tibeticola* | Genus |  |  |
| *Tissierella* | Genus |  |  |
| *Trichococcus* | Genus |  |  |
| *Truepera* | Genus |  |  |
| *Tsukamurella* | Genus |  |  |
| *Tuberibacillus* | Genus |  |  |
| *Tumebacillus* | Genus |  |  |
| *Tunicatimonas* | Genus |  |  |
| *Turicibacter* | Genus |  |  |
| *Tyzzerella* | Genus |  |  |
| UBA6140 | N/A^3^ |  |  |
| UCG-002 | N/A^3^ |  |  |
| UCG-005 | N/A^3^ |  |  |
| UCG-012 | N/A^3^ |  |  |
| UTBCD1 | N/A^3^ |  |  |
| *Uliginosibacterium* | Genus |  |  |
| *Ulvibacter* | Genus |  |  |
| Unclassified ASVs^4^ | Unclassified^4^ |  |  |
| *Undibacterium* | Genus |  |  |
| *Ureibacillus* | Genus |  |  |
| *Vagococcus* | Genus |  |  |
| *Variibacter* | Genus |  |  |
| *Variovorax* | Genus |  |  |
| *Verrucosispora* | Genus |  |  |
| *Verticiella* | Genus |  |  |
| *Vicingus* | Genus |  |  |
| *Virgibacillus* | Genus |  |  |
| *Virgisporangium* | Genus |  |  |
| *Vogesella* | Genus |  |  |
| *Vulgatibacter* | Genus |  |  |
| WCHB1-32 | N/A^3^ |  |  |
| *Weissella* | Genus |  |  |
| *Wenxinia* | Genus |  |  |
| *Williamsia* | Genus |  |  |
| *Woeseia* | Genus |  |  |
| *Wolbachia* | Genus |  |  |
| *Xanthomonas* | Genus |  |  |
| *Xylophilus* | Genus |  |  |
| YC-ZSS-LKJ147 | N/A^3^ |  |  |
| *Yersinia* | Genus |  |  |
| *Youngiibacter* | Genus |  |  |
| *Zoogloea* | Genus |  |  |
| *Zymobacter* | Genus |  |  |
| [Eubacterium] hallii group | N/A^3^ |  |  |
| [Eubacterium] tenue group | N/A^3^ |  |  |
| [Renibacterium] salmoninarum group | N/A^3^ |  |  |
| [Rhizobium] sphaerophysae group | N/A^3^ |  |  |
| [Ruminococcus] torques group | N/A^3^ |  |  |
| alphaI cluster | N/A^3^ |  |  |
| dgA-11 gut group | N/A^3^ |  |  |
| hgcI clade | N/A^3^ |  |  |
| mle1-7 | N/A^3^ |  |  |
| possible genus 04 | N/A^3^ |  |  |
| s3t2d-1089 | N/A^3^ |  |  |

^1^The groups in this column were from the “genus” field in the taxonomic table produced as an output when the Amplicon Sequence Variants (ASVs) were queried against the SILVA 16S rRNA database v. 138.1.

^2^The groups in this column were from the “genus” field in the taxonomic table produced as an output when the Operational Taxonomic Units (OTUs) were queried against the UNITE fungal database v. 10.0. OTUs that were classified at a higher taxonomic level (e.g. family) were assigned the correct taxonomic level in this column.

^3^Bacterial ASVs and Fungal OTUs that could not be classified into a taxonomic group were grouped as “Unclassified ASVs” and “Unclassified OTUs”, respectively.

^4^ N/A means Not Applicable; these groups were not recognized as a genus by BacDive (https://bacdive.dsmz.de/), a database for standardized bacterial taxonomic information.

Supplementary Table 3. The top five bacterial and fungal groups in terms of average relative abundance of all Salinas, CA and Yuma, AZ area samples at Harvest, Day Initial and two key days of shelf life.

| **Rank** | **Harvest (Yuma, AZ/Salinas, CA areas)**^1^ | **Day Initial (Yuma, AZ/Salinas, CA areas)**^1^ | **Day 7 (Yuma, AZ/Salinas, CA areas)**^1^ | **Day 21/22 (Yuma, AZ/Salinas, CA areas)**^1^ |
| --- | --- | --- | --- | --- |
| *Bacterial Groups* | | | | |
| 1 | *Buchnera/Bacillus* (22.8%/14.8%) | *Pseudomonas* (78.1/56.9%) | *Pseudomonas* (71.2%/77.1%) | *Pseudomonas* (78.1%/56.9%) |
| 2 | Unclassified ASVs  (13.8%/12.5%) | *Flavobacterium*  (5.1%/15.5%) | *Pantoea*  (10.0%/4.8%) | *Flavobacterium*  (5.1%/15.5%) |
| 3 | *Pantoea/Pseudomonas* (9.9%/9.1%) | *Pantoea/ Shewanella* (4.2%/5.0%) | *Shewanella/Erwinia*  (5.9%/3.7%) | *Pantoea*  */Shewanella*  (4.2%/5.0%) |
| 4 | *Pseudomonas/Pantoea* (7.9%/8.4%) | *Duganella/Sphingobacterium* (2.9%/3.4%) | *Psychrobacter/Exiguobacterium* (3.8%/2.5%) | *Duganella/Sphingobacterium*  (2.9%/3.5%) |
| 5 | *Bacillus/Allorhizobium-Neorhizobium-Pararhizobium-Rhizobium* (6.4%/7.4%) | *Shewanella/Pantoea*  (2.4%/3.3%) | *Duganella/ Psychrobacter*  (1.3%/2.1%) | *Shewanella*/*Pantoea*  (2.4%/3.3%) |
| Sum of Top Five Groups | 60.8%/52.2% | 92.7%/84.1% | 92.2%/90.2% | 92.7%/84.2% |
| *Fungal Groups* | | | | |
| 1 | *Alternaria/Cladosporium* (28.1%/28.3%) | *Cladosporium* (32.2%/38.6%) | *Cladosporium* (30.8%/31.6%) | *Cladosporium*  (27.2%/38.8%) |
| 2 | *Cladosporium/Alternaria*  (27.2 %/16.2%) | *Alternaria*  (30.5%/14.3%) | *Alternaria/Vishniacozyma* (27.1%/28.1%) | *Alternaria/Vishniacozyma*  (25.1%/23.8%) |
| 3 | Unclassified *Pleosporales/*Unclassified OTUs (5.4%/13.7) | *Vishniacozyma* (13.1%/13.7%) | *Vishniacozyma/Alternaria*  (17.2%/10.9%) | *Vishniacozyma*/Unclassified  *Pleosporales*  (23.6%/12.0%) |
| 4 | *Stemphylium* (4.7%/12.0%) | Unclassified *Pleosporales/ Stemphylium*  (5.5%/9.2%) | *Cryptococcus*/Unclassified *Pleosporales*  (7.6%/7.3%) | Cryptococcus*/Stemphylium*  (11.2%/10.6%) |
| 5 | *Curvularia/Vishniacozyma* (4.6%/6.0%) | *Stemphylium/* Unclassified *Pleosporales*  (4.0%/8.2%) | Unclassified *Pleosporales/Stemphylium*  (5.2%/7.1%) | Unclassified  *Pleosporales/Alternaria*  (7.0%/10.0%) |
| Sum of the Top Five Groups | 70.0%/76.2% | 85.3%/84.0% | 87.9%/85.0% | 94.1%/95.2% |

^1^ These percentages were calculated using all samples from the Yuma, AZ and Salinas, CA areas; SE USA samples (FL and GA) are not included in the data shown here.

Supplementary Table 4. Differentially abundant bacterial groups in Harvest samples by area^1^

| **Groups** | **Average Relative Abundance (%)**^2^ **in** | | **Log_2_ Fold Change^3^** | **Significance Level^4^** |
| --- | --- | --- | --- | --- |
|  | **Salinas, CA area** | **Yuma, AZ area** |  |  |
| ***Groups that are significantly enriched in the Salinas, CA area*** | | | | |
| *Qipengyuania* | 0.2 | 0.01 | -4.9 | ** |
| *Gaiella* | 0.1 | 0.0 | -4.7 | * |
| *Streptomyces* | 0.6 | 0.06 | -3.4 | ** |
| *Paracoccus* | 3.4 | 0.4 | -3.00 | ** |
| *Lautropia* | 0.1 | 0.01 | -2.9 | * |
| *Amaricoccus* | 0.2 | 0.03 | -2.6 | * |
| *Allorhizobium-Neorhizobium-Pararhizobium-Rhizobium* | 7.0 | 1.2 | -2.5 | * |
| *Nitrospira* | 0.6 | 0.11 | -2.4 | * |
| *Terribacillus* | 1.3 | 0.3 | -2.2 | ** |
| *MND1* | 0.2 | 0.04 | -2.00 | * |
| *Lysobacter* | 0.8 | 0.2 | -2.00 | * |
| *Nocardioides* | 1.1 | 0.3 | -1.9 | * |
| *Bacillus* | 14.8 | 6.8 | -1.1 | * |
| ***Groups that are significantly enriched in Yuma, AZ area*** | | | | |
| *Lysinibacillus* | 0.1 | 0.3 | 1.7 | * |
| *Clostridium sensu stricto 13* | 0.07 | 0.2 | 1.8 | ** |
| *Flavobacterium* | 0.2 | 1.2 | 2.6 | ** |
| *Tumebacillus* | 0.1 | 0.9 | 3.2 | ** |
| *Sphingoaurantiacus* | 0.01 | 0.09 | 3.9 | ** |
| *Ammoniphilus* | 0.07 | 1.0 | 3.9 | *** |
| *Clostridium sensu stricto 10* | 0.01 | 0.1 | 4.0 | *** |
| *Geodermatophilus* | 0.04 | 0.7 | 4.0 | ** |
| *Clostridium sensu stricto 1* | 0.07 | 1.2 | 4.1 | *** |
| *Romboutsia* | 0.05 | 1.3 | 4.5 | ** |
| *Brevibacillus* | 0.01 | 0.3 | 5.1 | ** |
| *Clostridium sensu stricto 8* | 0.02 | 0.8 | 5.2 | *** |
| *Turicibacter* | 0.01 | 0.7 | 5.7 | ** |
| *Virgibacillus* | 0.00 | 0.5 | 5.7 | ** |
| *Buchnera* | 0.4 | 22.8 | 5.8 | *** |
| *Archangium* | 0.00 | 0.1 | 5.9 | * |
| *Rhodoferax* | 0.00 | 0.2 | 6.0 | ** |
| *Hydrogenophaga* | 0.00 | 0.2 | 6.6 | * |
| *Paeniclostridium* | 0.00 | 0.2 | 7.0 | * |
| *Fonticella* | 0.00 | 0.2 | 7.3 | ** |

^1^ Only Salinas, CA and Yuma, AZ area samples were included in this analysis, for a total of 20 Harvest samples.

^2^ Rarefied read counts were used to calculate relative abundance, and the relative abundance was used as the input data for this analysis. The following settings were used in the MaAsLin2 code for this analysis: analysis_method= CPLM, min_prevalence = 0, min_abundance = 0.

^3^ Log_2_ Fold Change was calculated using the model coefficient, which is part of MaAsLin2 output. The coefficient, which is in natural log, was exponentiated, and then log_2_ transformed (the R code was Log_2_ Fold Change=log2(exp(coef))). Average relative abundance is provided as a reference but was calculated directly from the same data used as MaAsLin2 input, not MaAsLin2 output.

^4^ * refers to p<0.05; **p<0.01; ***p<0.001.A dash ‘-‘ indicates no evidence for significant differential abundance. Significance level was derived from the False Discovery Rate.Supplementary Table 5. Differentially abundant bacterial groups in Harvest samples by days from the start of the planting season^1^

| **Groups** | **Relative Abundance (%)**^2^ **for the** | | **Log_2_ Fold Change^3^** | **Significance Level^4^** |
| --- | --- | --- | --- | --- |
|  | **First Half of Planting Season** | **Second Half of Planting Season** |  |  |
| *Ralstonia* | 0.1 | 0.0 | -11.3 | * |
| *OM27 clade* | 0.06 | 0.04 | -2.4 | * |
| *Pedobacter* | 0.08 | 0.01 | -1.7 | *** |
| *Ammoniphilus* | 0.7 | 0.2 | -1.4 | *** |
| *Rubellimicrobium* | 0.7 | 0.1 | -1.3 | ** |
| *Flaviaesturariibacter* | 0.1 | 0.02 | -1.2 | * |
| *Clostridium sensu stricto 13* | 0.2 | 0.04 | -1.2 | *** |
| *Haliangium* | 0.1 | 0.03 | -1.2 | ** |
| *Flavisolibacter* | 0.3 | 0.07 | -1.2 | ** |
| *Kocuria* | 0.2 | 0.03 | -1.2 | ** |
| *Pontibacter* | 0.2 | 0.05 | -1.1 | *** |
| *Noviherbaspirillum* | 0.5 | 0.1 | -1.1 | *** |
| *Salinicoccus* | 1.1 | 0.2 | -1.1 | * |
| *Gemmatimonas* | 0.1 | 0.03 | -1.1 | * |
| *Altererythrobacter* | 0.2 | 0.02 | -1.1 | * |
| *Clostridium sensu stricto 1* | 0.7 | 0.4 | -1.0 | ** |
| *Devosia* | 0.2 | 0.06 | -0.9 | ** |
| *Microlunatus* | 0.3 | 0.08 | -0.9 | ** |
| *Lysinibacillus* | 0.3 | 0.1 | -0.9 | ** |
| *Lysobacter* | 0.8 | 0.3 | -0.8 | ** |
| *Ramlibacter* | 0.2 | 0.06 | -0.8 | * |
| *Microvirga* | 1.1 | 0.4 | -0.8 | ** |
| *Blastococcus* | 0.4 | 0.1 | -0.8 | * |
| *Skermanella* | 0.7 | 0.2 | -0.9 | *** |
| *Massilia* | 2.0 | 0.6 | -0.8 | ** |
| *Pseudarthrobacter* | 0.2 | 0.06 | -0.8 | * |
| *Sphingomonas* | 2.5 | 0.9 | -0.8 | *** |
| *Steroidobacter* | 0.3 | 0.10 | -0.7 | * |
| *MND1* | 0.2 | 0.08 | -0.7 | * |
| Unclassified ASVs | 17.2 | 7.2 | -0.5 | * |

^1^ Only Salinas, CA and Yuma, AZ area samples were included in this analysis, for a total of 20 Harvest samples.

^2^ Rarefied read counts were used to calculate relative abundance, and the relative abundance was used as the input data for this analysis. The following settings were used in the MaAsLin2 code for this analysis: analysis_method= CPLM, min_prevalence = 0, min_abundance = 0.

^3^ Log_2_ Fold Change was calculated using the model coefficient, which is part of MaAsLin2 output. The coefficient, which is in natural log, was exponentiated, and then log_2_ transformed (the R code was Log_2_ Fold Change=log2(exp(coef))). Average relative abundance is provided as a reference but was calculated directly from the same data used as MaAsLin2 input, not MaAsLin2 output.

^4^ * refers to p<0.05; **p<0.01; ***p<0.001. Significance level was derived from the False Discovery Rate.Supplementary Table 6. PERMANOVA and PERMDISP p-values for different sample sets and Day and Area variables^1^

| **Samples and Variables (Day and/or Area)** | **p-value** | |
| --- | --- | --- |
|  | **16S rRNA samples** | **ITS samples** |
| Harvest samples; Area (PERMANOVA) | 0.001 | 0.001 |
| Harvest samples; Area (PERMDISP) | 0.01 | 0.002 |
| Harvest and DI samples; Interaction of Day and Area | 0.001 | 0.001 |
| DI samples; Area (PERMANOVA) | 0.1 | 0.1 |
| 5-day interval packaged samples; Day | 0.005 | 0.005 |
| 5-day interval packaged samples; Area | 0.005 | 0.005 |
| 5-day interval packaged samples; Interaction of Day and Area | 0.2 | 0.9 |
| 7-day interval packaged samples; Day | 0.005 | 0.005 |
| 7-day interval packaged samples; Area | 0.005 | 0.005 |
| 7-day interval packaged samples; Interaction of Day and Area | 1.0 | 1.0 |

^1^ Only Salinas, CA and Yuma, AZ area samples were included in this analysis, for a total of 20 Harvest samples, 22 DI samples and 22 packaged sample sets.

Supplementary Table 7. Alpha diversity index values for Harvest samples (by Area and number of days from the start of the planting season) and Harvest and DI samples (by Day)^1^

| **Alpha Diversity Indices** |  | | **Harvest samples** | | | | |  | **Harvest and DI samples** | | |
| --- | --- | --- | --- | --- | --- | --- | --- | --- | --- | --- | --- |
|  | **Area** | | | |  | **Number of days after the start of the planting season** | |  | **Day** | | |
|  | **Yuma, AZ area** | **Salinas, CA area** | | **Significance** |  | **Correlation Value** | **Significance** |  | **H** | **DI** | **Significance** |
| *Bacterial* |  |  | |  |  |  |  |  |  |  |  |
| **Average Pielou’s (Evenness)** | 0.65 | 0.77 | | * | | -0.37 | - |  | 0.73 | 0.65 | *** |
| **Average Shannon Index** | 3.92 | 4.55 | | - | | -0.65 | *** |  | 4.29 | 3.12 | *** |
| **Average Richness** | 411.5 | 399.625 | | - | | -0.74 | *** |  | 397.17 | 131.96 | *** |
| *Fungal* |  |  | |  |  |  |  |  |  |  |  |
| **Average Pielou’s (Evenness)** | 0.51 | 0.52 | | - | | -0.098 | - |  | 0.52 | 0.47 | ** |
| **Average Shannon Index** | 2.27 | 2.33 | | - | | -0.23 | - |  | 2.32 | 1.86 | *** |
| **Average Richness** | 83.81 | 93.25 | | - | | -0.53 | * |  | 93.55 | 54.74 | *** |

^1^ Only Salinas, CA and Yuma, AZ area samples were included in this analysis, for a total of 20 Harvest and 22 DI samples.

Supplementary Table 8. Bacterial groups enriched by area from packaged samples^1^

| **Groups** | **Average Relative Abundance (%)**^2^ **in** | | **Log_2_ Fold Change^3^** | **Significance Level^4^** |
| --- | --- | --- | --- | --- |
|  | **Yuma, AZ area** | **Salinas, CA area** |  |  |
| ***Groups that are significantly enriched in Salinas, CA area*** | | | | |
| *Lelliottia* | 0.01 | 0.06 | -3.8 | *** |
| *Stenotrophomonas* | 0.3 | 1.1 | -2.8 | ** |
| *Chryseobacterium* | 0.2 | 1.1 | -2.2 | ** |
| ***Groups that are significantly enriched in Yuma, AZ area*** | | | | |
| *Clostridium sensu stricto 10* | 0.01 | 0.0 | 3.3 | * |
| *Ammoniphilus* | 0.03 | 0.0 | 4.2 | ** |
| *Clostridium sensu stricto 1* | 0.06 | 0.02 | 4.3 | * |
| *Clostridium sensu stricto 8* | 0.04 | 0.00 | 5.1 | *** |
| *Romboutsia* | 0.1 | 0.02 | 6.6 | * |

^1^ Only Salinas, CA and Yuma, AZ area samples were included in this analysis, for a total of 22 packaged sample sets.

^2^ Rarefied read counts were used to calculate relative abundance, and the relative abundance was used as the input data. The following settings were used in the MaAsLin2 code for this analysis: analysis_method= CPLM, min_prevalence = 0, min_abundance = 0.

^3^ Log_2_ Fold Change was calculated using the model coefficient, which is part of MaAsLin2 output. The coefficient, which is in natural log, was exponentiated, and then log_2_ transformed (the R code was Log_2_ Fold Change=log2(exp(coef))). Average relative abundance is provided as a reference but was calculated directly from the same data used as MaAsLin2 input, not MaAsLin2 output.

^4^ * refers to p<0.05; **p<0.01; ***p<0.001. Significance level was derived from the False Discovery Rate.

Supplementary Table 9. Bacterial ASVs Differentially Abundant in Packaged Samples over Shelf Life and by Area^1^

| **Groups of Enriched ASVs** | **Total Number of ASVs assigned to Groups**^2^ | **Number of ASVs Differentially Abundant by:** | | | | |
| --- | --- | --- | --- | --- | --- | --- |
|  |  | **Area** | |  | **Over Shelf Life** | |
|  |  | **Salinas, CA area** | **Yuma, AZ area** |  | **Decreasing^3^** | **Increasing** |
| *Pseudomonas* | 868 | 12 | 3 |  | 23 | 39 |
| *Flavobacterium* | 387 | 1 | 0 |  | 0 | 32 |
| *Bacillus* | 267 | 0 | 0 |  | 4 | 0 |
| *Massilia* | 237 | 0 | 0 |  | 5 | 0 |
| *Sphingomonas* | 125 | 0 | 0 |  | 1 | 0 |
| *Shewanella* | 120 | 0 | 0 |  | 0 | 9 |
| *Pantoea* | 91 | 0 | 0 |  | 9 | 0 |
| *Duganella* | 77 | 0 | 0 |  | 0 | 7 |
| *Allorhizobium-Neorhizobium-Pararhizobium-Rhizobium* | 74 | 0 | 0 |  | 2 | 1 |
| *Chryseobacterium* | 68 | 1 | 0 |  | 0 | 3 |
| *Exiguobacterium* | 66 | 2 | 0 |  | 5 | 0 |
| *Stenotrophomonas* | 65 | 4 | 0 |  | 0 | 5 |
| *Planomicrobium* | 62 | 0 | 0 |  | 4 | 0 |
| *Sphingobacterium* | 60 | 1 | 0 |  | 0 | 4 |
| *Psychrobacter* | 59 | 0 | 0 |  | 2 | 0 |
| *Buchnera* | 53 | 0 | 0 |  | 1 | 0 |
| *Planococcus* | 49 | 0 | 0 |  | 3 | 0 |
| *Janthinobacterium* | 44 | 0 | 0 |  | 0 | 7 |
| *Clostridium sensu stricto 1* | 42 | 0 | 0 |  | 1 | 0 |
| *Paracoccus* | 23 | 0 | 0 |  | 1 | 0 |
| *Carnobacterium* | 20 | 0 | 0 |  | 1 | 0 |
| *Rahnella1* | 17 | 0 | 0 |  | 0 | 2 |
| *Acidovorax* | 14 | 0 | 0 |  | 0 | 1 |
| *Turicibacter* | 13 | 0 | 0 |  | 1 | 0 |
| *Oxalicibacterium* | 13 | 0 | 0 |  | 0 | 3 |
| *Buttiauxella* | 10 | 0 | 0 |  | 0 | 2 |
| *Rugamonas* | 8 | 0 | 0 |  | 0 | 1 |
| *Terribacillus* | 6 | 0 | 0 |  | 1 | 0 |
| *Lelliottia* | 6 | 1 | 0 |  | 0 | 0 |
| *Romboutsia* | 5 | 0 | 1 |  | 1 | 0 |

1 Only Salinas, CA and Yuma, AZ area samples were included in this analysis, for a total of 22 packaged sample sets.

^2^ Rarefied read counts were used as the input data for MaAsLin2. The following settings were used in the MaAsLin2 code for this analysis: analysis_method= CPLM, min_prevalence = 0.1, min_abundance = 0.01. All reported ASVs had a significant differential abundance (p<0.05).

^3^ ‘Decreasing’ refers to ‘significantly decreasing in differential abundance’ and ‘increasing’ refers to ‘significantly increasing in differential abundance’.

Supplementary Table 10. Average alpha diversity indices for each five-day and seven-day timepoints over sample shelf life for bacterial and fungal communities from Salinas, CA and Yuma, AZ area samples

| **Sample** | **Day** | **Average Pielou’s (Evenness)** ^1,2^ | **Average Shannon Index** | **Average Richness** |
| --- | --- | --- | --- | --- |
| Bacterial | *Five Day Interval* | | | |
|  | Day Initial | 0.65^AB^ | 3.20^AB^ | 144.91^A^ |
|  | Day 7 | 0.64^A^ | 2.94^A^ | 101.03^B^ |
|  | Day 12 | 0.70^B^ | 3.30^B^ | 119.34^AB^ |
|  | Day 17 | 0.69^B^ | 3.33^B^ | 128.03^AB^ |
|  | Day 22 | 0.69^B^ | 3.33^B^ | 129.06^A^ |
|  | *Seven Day Interval* | | | |
|  | Day Initial | 0.63^A^ | 2.88^A^ | 95.75^A^ |
|  | Day 7 | 0.70^B^ | 3.20^AB^ | 98.92^A^ |
|  | Day 14 | 0.72^B^ | 3.52^BC^ | 136.00^B^ |
|  | Day 21 | 0.74^B^ | 3.64^C^ | 140.00^B^ |
|  | Day 28 | 0.74^B^ | 3.72^C^ | 148.83^B^ |
|  |  |  |  |  |
| Fungal | *Five Day Interval* | | | |
|  | Day Initial | 0.60^A^ | 3.24^A^ | 271.24^A^ |
|  | Day 7 | 0.60^AB^ | 2.98^AB^ | 156.70^AB^ |
|  | Day 12 | 0.66^B^ | 3.33^C^ | 172.06^BC^ |
|  | Day 17 | 0.65^AB^ | 3.36^BC^ | 193.86^C^ |
|  | Day 22 | 0.65^AB^ | 3.36 ^C^ | 193.94^C^ |
|  | *Seven Day Interval* | | | |
|  | Day Initial | 0.46^A^ | 1.83^A^ | 56.79^A^ |
|  | Day 7 | 0.45^A^ | 1.70^A^ | 44.79^AB^ |
|  | Day 14 | 0.46^A^ | 1.60^A^ | 34.80^BC^ |
|  | Day 21 | 0.49^A^ | 1.67^A^ | 29.38^BC^ |
|  | Day 28 | 0.49^A^ | 1.60^A^ | 26.33^C^ |

^1^ via ANOVA and post hoc Tukey test.

^2^ Different superscript uppercase letters in the same column indicate a significant difference (p < 0.05) between different days for a given alpha diversity index. Samples were divided by the timeline they were tested with, either in a 5-day interval or 7-day interval.

Supplementary Table 11. Differentially abundant fungal groups in Harvest samples by area^1^

| **Groups** | **Average Relative Abundance**^2^ **(%) in** | | | **Log_2_ Fold Change^3^** | **Significance Level^4^** |
| --- | --- | --- | --- | --- | --- |
|  | **Yuma, AZ area** | **Salinas, CA area** | |  |  |
| ***Groups that are significantly enriched in the Salinas, CA area*** | | | | | |
| *Malbranchea* | 0.12 | 0.8 | -4.0 | | ** |
| *Fusarium* | 0.3 | 2.2 | -3.1 | | * |
| *Penicillium* | 0.05 | 0.6 | -3.6 | | *** |
| Unclassified OTUs | 2.5 | 13.7 | -2.5 | | * |
| ***Groups that are significantly enriched in Yuma, AZ area*** | | | | | |
| *Curvularia* | 4.6 | 0.0 | 12.3 | | *** |
| *Canariomyces* | 0.4 | 0.0 | 8.7 | | ** |
| *Celosporium* | 0.6 | 0.0 | 7.9 | | * |
| *Knufia* | 0.5 | 0.01 | 6.0 | | * |
| *Stachybotrys* | 0.04 | 0.03 | 4.0 | | *** |

^1^ Only Salinas, CA and Yuma, AZ area samples were included in this analysis, for a total of 20 Harvest samples.

^2^ Rarefied read counts were used to calculate relative abundance, and the relative abundance was used as the input data for this analysis. The following settings were used in the MaAsLin2 code for this analysis: analysis_method= CPLM, min_prevalence = 0, min_abundance = 0.

^3^ Log_2_ Fold Change was calculated using the model coefficient, which is part of MaAsLin2 output. The coefficient, which is in natural log, was exponentiated, and then log_2_ transformed (the R code was Log_2_ Fold Change=log2(exp(coef))). Average relative abundance is provided as a reference but was calculated directly from the same data used as MaAsLin2 input, not MaAsLin2 output.

^4^ * refers to p<0.05; **p<0.01; ***p<0.001. Significance level was derived from the False Discovery Rate.Supplementary Table 12. Differentially abundant fungal groups in packaged samples by area^1^

| **Groups** | **Average Relative Abundance (%)**^2^ **in** | | **Log_2_ Fold Change^3^** | **Significance Level^4^** |
| --- | --- | --- | --- | --- |
|  | **Yuma, AZ area** | **Salinas, CA area** |  |  |
| ***Groups that are significantly enriched in the Salinas, CA area*** | | | | |
| Unclassified *Hypocreales* | 0.004 | 0.3 | -7.2 | *** |
| *Bisifusarium* | 0.0003 | 0.03 | -6.1 | * |
| *Neodevriesia* | 0.0003 | 0.01 | -4.9 | * |
| *Botrytis* | 0.2 | 3.1 | -3.8 | ** |
| *Malbranchea* | 0.01 | 0.1 | -3.6 | *** |
| *Symmetrospora* | 0.00 | 0.04 | -3.00 | ** |
| *Fusarium* | 0.06 | 0.2 | -2.9 | * |
| Unclassified *Basidiomycota* | 0.01 | 0.03 | -2.4 | ** |
| *Stemphylium* | 2.44 | 8.7 | -1.5 | * |
| ***Groups that are significantly enriched in the Yuma, AZ area*** | | | | |
| *Acremonium* | 0.03 | 0.02 | 1.2 | *** |
| *Alternaria* | 27.8 | 11.7 | 1.5 | * |
| Unclassified *Sordariales* | 0.01 | 0.001 | 2.4 | * |
| *Gymnoascus* | 0.003 | 0.0004 | 3.2 | * |
| *Tulostoma* | 0.01 | 0.0006 | 3.4 | * |
| Unclassified *Dothideomycetes* | 0.12 | 0.004 | 3.7 | ** |
| *Preussia* | 0.09 | 0.01 | 3.7 | ** |
| *Knufia* | 0.03 | 0.003 | 3.9 | * |
| Unclassified *Myriangiales* | 0.01 | 0.0004 | 4.1 | * |
| *Sporormiella* | 0.02 | 0.001 | 4.2 | * |
| *Staurosphaeria* | 0.03 | 0.0008 | 4.6 | * |
| Unclassified *Sordariomycetes* | 0.01 | 0.0002 | 4.6 | * |
| *Celosporium* | 0.04 | 0.001 | 5.0 | ** |
| *Neocamarosporium* | 0.16 | 0.002 | 5.3 | * |
| *Toxicocladosporium* | 0.01 | 0.0002 | 6.12 | ** |
| *Dematiopleospora* | 0.07 | 0.0006 | 7.7 | * |
| *Canariomyces* | 0.06 | 0.003 | 7.89 | * |
| *Allocanariomyces* | 0.1 | 0.003 | 9.6 | ** |
| *Curvularia* | 0.6 | 0.05 | 10.7 | ** |
| *Bipolaris* | 0.2 | 0.2 | 12.5 | * |

^1^ Only Salinas, CA and Yuma, AZ area samples were included in this analysis for a total of 22 packaged sample sets.

^2^ Rarefied read counts were used to calculate relative abundance, and the relative abundance was used as the input data. The following settings were used in the MaAsLin2 code for this analysis: analysis_method= CPLM, min_prevalence = 0, min_abundance = 0.

^3^ Log_2_ Fold Change was calculated using the model coefficient, which is part of MaAsLin2 output. The coefficient, which is in natural log, was exponentiated, and then log_2_ transformed (the R code was Log_2_ Fold Change=log2(exp(coef))). Average relative abundance is provided as a reference but was calculated directly from the same data used as MaAsLin2 input, not MaAsLin2 output.

^4^ * refers to p<0.05; **p<0.01; ***p<0.001. Significance level was derived from the False Discovery Rate.

Supplementary Table 13. Fungal OTUs Differentially Abundant in Packaged samples over Shelf Life and by Area^1^

| **Groups** | **Total Number of OTUs assigned to Groups^2^** | **Number of OTUs Differentially Abundant by:** | | | | |
| --- | --- | --- | --- | --- | --- | --- |
|  |  | **Area** | |  | **Over Shelf Life** | |
|  |  | **Salinas, CA area** | **Yuma, AZ area** |  | **Increasing** | **Decreasing** |
| Unclassified OTUs | 82 | 0 | 1 |  | 0 | 4 |
| *Alternaria* | 35 | 1 | 3 |  | 1 | 3 |
| *Penicillium* | 29 | - | - |  | 0 | 2 |
| Unclassified *Ascomycota* | 28 | - | - |  | 0 | 2 |
| Unclassified *Pleosporales* | 27 | - | - |  | 1 | 1 |
| *Aspergillus* | 24 | 0 | 1 |  | 0 | 2 |
| *Curvularia* | 19 | 0 | 1 |  | 0 | 1 |
| *Fusarium* | 16 | 1 | 0 |  | 0 | 4 |
| *Acremonium* | 11 | - | - |  | 0 | 3 |
| Unclassified *Agaricales* | 10 | - | - |  | 0 | 1 |
| *Vishniacozyma* | 10 | - | - |  | 2 | 0 |
| Unclassified *Polyporales* | 9 | - | - |  | 0 | 1 |
| *Candida* | 8 | - | - |  | 0 | 2 |
| Unclassified *Pleosporaceae* | 8 | - | - |  | 0 | 1 |
| *Comoclathris* | 7 | 0 | 1 |  | 0 | 2 |
| *Unclassified Hypocreales* | 7 | 1 | 0 |  | 1 | 0 |
| *Symmetrospora* | 7 | 1 | 0 |  | 0 | 1 |
| Unclassified *Basidiomycota* | 6 | 1 | 0 |  | 1 | 0 |
| *Golovinomyces* | 6 | - | - |  | 0 | 1 |
| *Bipolaris* | 5 | 0 | 1 |  | 0 | 2 |
| *Erysiphe* | 5 | - | - |  | 0 | 1 |
| *Neodevriesia* | 5 | 1 | 0 |  | 0 | 1 |
| *Aureobasidium* | 4 | 0 | 1 |  | 0 | 2 |
| *Cryptococcus* | 4 | - | - |  | 2 | 0 |
| *Sporobolomyces* | 4 | 0 | 1 |  | 0 | 2 |
| Unclassified *Sporormiaceae* | 4 | - | - |  | 0 | 1 |
| Unclassified *Dothideales* | 3 | - | - |  | 0 | 1 |
| *Knufia* | 3 | 0 | 1 |  | 0 | 1 |
| *Leucosporidium* | 3 | - | - |  | 1 | 0 |
| *Neocamarosporium* | 3 | - | - |  | 0 | 2 |
| *Podosphaera* | 3 | - | - |  | 0 | 1 |
| *Stemphylium* | 3 | 3 | 0 |  | - | - |
| *Canariomyces* | 2 | 0 | 1 |  | 0 | 1 |
| *Cephaliophora* | 2 | - | - |  | 0 | 1 |
| *Chrysosporium* | 2 | - | - |  | 0 | 1 |
| *Cladosporium* | 2 | - | - |  | 0 | 1 |
| *Dematiopleospora* | 2 | 0 | 1 |  | 0 | 1 |
| *Exserohilum* | 2 | - | - |  | 0 | 1 |
| *Malbranchea* | 2 | 1 | 0 |  | 0 | 1 |
| Unclassified *Myriangiales* | 2 | 0 | 1 |  | 0 | 1 |
| *Rhizopus* | 2 | - | - |  | 0 | 1 |
| *Stachybotrys* | 2 | - | - |  | 0 | 1 |
| *Actinomucor* | 1 | - | - |  | 0 | 1 |
| *Allocanariomyces* | 1 | 0 | 1 |  | 0 | 1 |
| *Arxotrichum* | 1 | - | - |  | 0 | 1 |
| *Bezerromyces* | 1 | - | - |  | 0 | 1 |
| *Bisifusarium* | 1 | 1 | 0 |  | 0 | 1 |
| *Botryotrichum* | 1 | - | - |  | 0 | 1 |
| *Botrytis* | 1 | 1 | 0 |  | 0 | 1 |
| *Butlerelfia* | 1 | - | - |  | 0 | 1 |
| *Calvatia* | 1 | - | - |  | 0 | 1 |
| *Celosporium* | 1 | 0 | 1 |  | 0 | 1 |
| *Cercospora* | 1 | - | - |  | 0 | 1 |
| *Claviceps* | 1 | - | - |  | 0 | 1 |
| *Coniophora* | 1 | - | - |  | 0 | 1 |
| *Cutaneotrichosporon* | 1 | - | - |  | 0 | 1 |
| *Davidiellomyces* | 1 | - | - |  | 0 | 1 |
| *Epicoccum* | 1 | - | - |  | 0 | 1 |
| *Gymnoascus* | 1 | - | - |  | 0 | 1 |
| *Leucothecium* | 1 | - | - |  | 0 | 1 |
| *Salinomyces* | 1 | - | - |  | 0 | 1 |
| *Sampaiozyma* | 1 | - | - |  | 1 | 0 |
| *Thermomyces* | 1 | 0 | 1 |  | - | - |
| *Toxicocladosporium* | 1 | 0 | 1 |  | 0 | 1 |
| *Verticillium* | 1 | - | - |  | 0 | 1 |

1 Only Salinas, CA and Yuma, AZ area samples were included in this analysis, for a total of 22 packaged sample sets.

^2^ Rarefied read counts were used as the input data for MaAsLin2. The following settings were used in the MaAsLin2 code for this analysis: analysis_method= CPLM, min_prevalence = 0.1, min_abundance = 0.01. All reported ASVs had a significant differential abundance (p<0.05).

Supplementary Table 14. Summary of the Samples Used in this Study

| **Sampling Number** | **Sample Lot^1^** | **Sample Type^2^** | **Area^3^** | **Testing Interval^4^** | **Included in our final data set?** |
| --- | --- | --- | --- | --- | --- |
| 1 | 1221_A | Harvest | FL | - | Yes |
|  | 1221_A | Packaged | FL | 5-day interval | Yes |
| 2 | 0122_A | Packaged | AZ | 5-day interval | Yes |
| 3 | 0122_B | Harvest | AZ | - | Yes |
|  | 0122_B | Packaged | AZ | 5-day interval | Yes |
| 4 | 0222_A | Harvest | AZ | - | Yes |
|  | 0222_A | Packaged | AZ | 5-day interval | Yes |
| 5 | 0222_B | Harvest | AZ | - | Yes |
|  | 0222_B | Packaged | AZ | 5-day interval | Yes |
| 6 | 0322_A | Harvest | AZ | - | Yes |
|  | 0322_A | Packaged | AZ | 5-day interval | Yes |
| 7 | 0322_B | Harvest | AZ | - | Yes |
|  | 0322_B | Packaged | AZ | 5-day interval | Yes |
| 8 | 0422_A | Harvest | AZ | - | Yes |
|  | 0422_A | Packaged | AZ | 7-day interval | Yes, except D28 |
| 9 | 0422_B | Harvest | CA | - | Yes |
|  | 0422_B | Packaged | CA | 7-day interval | Yes, except D21 and D28 |
| 10 | 0522_A | Harvest | CA | - | Yes |
|  | 0522_A | Packaged | CA | 7-day interval | Yes, except D14, D21 and D28 |
| 11 | 0522_B | Harvest | CA | - | Yes |
|  | 0522_B | Packaged | CA | 7-day interval | Yes |
| 12 | 0522_C | Harvest | CA | - | Yes |
|  | 0522_C | Packaged | CA | 7-day interval | Yes |
| 13 | 0622_A | Harvest | CA | - | Yes |
|  | 0622_A | Packaged | CA | 7-day interval | Yes |
| 14 | 0622_B | Harvest | CA | - | Yes |
|  | 0622_B | Packaged | CA | 5-day interval | Yes |
| 15 | 0722_A | Harvest | CA | - | Yes |
|  | 0722_A | Packaged | CA | 5-day interval | Yes |
| 16 | 0722_B | Harvest | CA | - | No |
| 17 | 0822_A | Harvest | CA | - | Yes |
|  | 0822_A | Packaged | CA | 5-day interval | Yes |
| 18 | 0822_B | Packaged | CA | 5-day interval | Yes |
| 19 | 0922_A | Harvest | CA | - | Yes |
|  | 0922_A | Packaged | CA | 5-day interval | Yes |
| 20 | 0922_B | Harvest | CA | - | Yes |
|  | 0922_B | Packaged | CA | 5-day interval | No |
| 21 | 1022_A | Harvest | CA | - | Yes |
|  | 1022_A | Packaged | CA | 5-day interval | Yes |
| 22 | 1022_B | Harvest | CA | - | Yes |
|  | 1022_B | Packaged | CA | 5-day interval | Yes |
| 23 | 1122_A | Packaged | CA | 5-day interval | Yes |
| 24 | 1122_B | Packaged | GA | 5-day interval | Yes, except DI |
| 25 | 1222_A | Harvest | AZ | - | Yes |
|  | 1222_A | Packaged | AZ | 5-day interval | Yes |
| 26 | 1222_B | Harvest | AZ | - | No |
|  | 1222_B | Packaged | AZ | 5-day interval | Yes |
| 27 | 1222_C | Harvest | AZ | - | Yes |

^1^ The sample lot name indicates month and year of sampling, e.g. 0122A denotes sampling in January (denoted by “01”) of 2022 (denoted by “22”) with A denoting the first sampling in a given month, B denoting the second sampling, etc.

^2^ The term “Packaged” designated a set of samples that were tested over shelf life from Day Initial through Day 22 or 28

^3^ FL= Florida; AZ = Yuma, Arizona and Imperial Valley, California area; CA= Salinas, California area and GA= Georgia

^4^ Packaged samples were either tested every 5 days (5-day interval, which includes days Initial [DI], 7 [D7], 12 [D12], 17 [D17], and 22 [D22] or every 7 days (7-day interval, which includes days DI, D7, D14, D21, and D28)

Supplementary Table 15. Breakdown of Samplings by Area

| **Area^1^** | **AZ** | **CA** | **GA** | **FL** | **Total** |
| --- | --- | --- | --- | --- | --- |
| Number of Harvest Samples | 8 | 12 | 0 | 1 | 21 |
| Number of Packaged Samples (Day Initial through Day 22 or 28) | 9 | 13 | 1 | 1 | 24 |
| Number of Harvest and Packaged samples from the same lot^2^ | 6 | 9 | 0 | 1 | 16^3^ |

^1^AZ = Yuma, Arizona and Imperial Valley, California area; CA= Salinas, California area; GA= Georgia and FL= Florida

^2^ For some samples, we were able to collect Harvest and packaged (commercially washed) product from the same order (i.e., lot) to have matched samples for characterization

^3^While 17 Harvest and Packaged samples were obtained from the same lot, one Harvest Sample (1222_B) was not sequenced, resulting in 16 paired Harvest and Packaged samples that were used for data analyses.
